# Supplementary material for: Structural and functional insights into the delivery of a bacterial Rhs pore-forming toxin to the membrane
Source: Nat Commun. 2023 Nov 28;14:7808. doi: 10.1038/s41467-023-43585-5 (PMC10684867; doi:10.1038/s41467-023-43585-5)
Supplement: Supplementary file 1 — Supplementary Information [file 41467_2023_43585_MOESM1_ESM.pdf]

SUPPLEMENTARY INFORMATION:

Structural and functional insights into the delivery of a bacterial Rhs pore-forming toxin to the membrane

Amaia González-Magaña[1,2#], Igor Tascón[1,3#], Jon Altuna-Alvarez[1], María Queralt-Martín[4], Jake Colautti[5], Carmen Velázquez[1,2], Maialen Zabala[1,2], Jessica Rojas-Palomino[4], Marité Cárdenas[1,3], Antonio Alcaraz[4], John C. Whitney[5], Iban Ubarretxena-Belandia[1,3*], David Albesa-Jové[1,2,3*]

[1] Instituto Biofisika (CSIC, UPV/EHU), Fundación Biofísica Bizkaia/Biofisika Bizkaia Fundazioa (FBB), 48940 Leioa, Spain.

[2] Departamento de Bioquímica y Biología Molecular, University of the Basque Country, 48940 Leioa, Spain.

[3] Ikerbasque, Basque Foundation for Science, 48013 Bilbao, Spain

[4] Laboratory of Molecular Biophysics, Department of Physics, University Jaume I, 12071 Castellón, Spain

[5] Department of Biochemistry and Biomedical Sciences, Michael DeGroote Institute for Infectious Disease Research, and David Braley Centre for Antibiotic Discovery, McMaster University, Hamilton, Canada.

[#] These authors contributed equally to this work

Running title: The *P. aeruginosa* effector Tse5 encapsulates and delivers its toxic cargo to the membrane

* To whom correspondence should be addressed: David Albesa-Jové or Iban Ubarretxena-Belandia, Instituto Biofisika (UPV/EHU, CSIC), Scientific Park of the University of the Basque Country, Leioa, E-48940, Spain; Phone: +34 94 601 5171;

E–mails: david.albesa@ehu.eus, ivan.ubarrechena@ehu.eus

TABLE OF CONTENTS

Supplementary Note 1: Bioinformatic analysis of Tse5 homologues 3

Supplementary Tables 5

Supplementary Table 1 | Cryo-EM data collection processing and model refinement statistics. 5

Supplementary Table 2 | Descriptive graph describing the type of protein-protein interactions between Tse5-NT and Tse5-Shell calculated using Mapiya online server [5]. 6

Supplementary Table 3 | Summary of Small-angle X-ray scattering data analysis for Tse5 and Tse5 variants. 7

Supplementary Table 4 | Strains and Plasmids used in this study. 8

Supplementary Table 5 | Residues lining the interior of the Tse5-Shell that diverge from the consensus sequence found in Tse5 homologues containing C-terminal toxic fragments of putative/known enzymatic functions. 10

Supplementary Table 6 | Residues lining the exterior of the Tse5-Shell that diverge from the consensus sequence found in Tse5 homologues containing C-terminal toxic fragments of putative/known enzymatic functions. 11

Supplementary Figures 13

Supplementary Figure 1 | Workflow for cryo-EM structure determination. 13

Supplementary Figure 2 | Cryo-EM Structural determination of Tse5. 14

Supplementary Figure 3 | Tse5-Shell encapsulates the pore-forming Tse5-CT toxin 16

Supplementary Figure 4 | Residues Ser32-Lys47 anchor Tse5-NT fragment to Tse5-Rhs fragment through protein-protein interactions. 17

Supplementary Figure 5 | Tse5 is sensible to the membrane potential directionality. 18

Supplementary Figure 6 | Phylogenetic analysis of C-terminal fragments found in Tse5 homologues. 19

Supplementary Figure 7 | Alignment of Tse5 with homologues containing C-terminal toxic fragments of putative/known enzymatic functions 20

Supplementary Figure 8 | Electrostatic surface potential of Tse5, Rhs1 and RhsA. 21

Supplementary Figure 9 | Protein-membrane interactions and protein stability during MD simulation 22

Supplementary Figure 10 | Tse5 sequence derived for structural and biophysical studies. 23

Supplementary Figure 11 | Uncropped and unedited SDS-gels 24

Supplementary Note 2: LC-ESI-MS report for Tse5 25

Supplementary Note 3: N-terminal sequencing report Tse5-NT 29

Supplementary Note 4: N-terminal sequencing report Tse5-Shell 37

Supplementary Note 5: N-terminal sequencing report Tse5-CT 45

Supplementary references 53

Supplementary Note 1: Bioinformatic analysis of Tse5 homologues

To further evaluate how Tse5 differs from other Rhs proteins that contain enzymatic C-terminal toxins, we carried out a bioinformatic analysis to search for Tse5 homologues with Foldseek [1], which performs structural alignment of Tse5 with millions of predicted AlphaFold structures and the full Protein Databank. Using a sequence identity threshold of 18.2%, and after duplicated or truncated sequences were deleted, we identified 33 homologues (Supplementary Data 1). The first insight from this search is that Tse5 has diverged substantially from other Rhs proteins, with the closest homologue having 26.5% sequence identity.

Next, we classify homologues based on putative C-terminal toxicities. We identified 8 homologues with predicted DNase/RNase activity, 4 with predicted ADP-ribosyltransferase activity, one with predicted peptidoglycan hydrolase, one with double-stranded DNA cytidine deaminase, and one with a putative colicin activity (see Supplementary Fig. 6 for phylogenetic analysis of C-terminal fragments found in Tse5 homologues).

We then aligned Tse5 with homologues containing C-terminal toxin fragments of putative/known enzymatic functions (Supplementary Fig. 7a). This alignment allows identification of conservation/divergence of residues lining the interior and the exterior of the Tse5-Shell (Supplementary Fig. 7b). To conduct this analysis, we first calculated a consensus sequence between aligned sequences, using a consensus threshold of 65% identity. Then, we search for residues in the Tse5-Shell that diverge from the consensus sequence.

This alignment allows the identification of conservation/divergence of residues lining the interior and the exterior of Tse5-Shell (Supplementary Fig. 6). This bioinformatic analysis points to regions on the inner and outer surface of Tse5-Shell that accumulate mutations and, therefore, could be relevant

for its specialisation. We identify 32 residues lining the interior of the Tse5 cocoon that diverge from the consensus sequence (Supplementary Table 5). Seven mutations introduce a negative-charged residue, three introduce a positive-charged residue, and 14 introduce small nonpolar residues. Furthermore, 8 mutations substitute a hydrophobic residue with a small nonpolar or charged residue. Overall, these mutations modulate the electrostatic inner-surface potential, which could be important for the specialisation of Tse5 towards encapsulating and delivering a hydrophobic pore-forming toxin. Nonetheless, given that only 27 residues of the Tse5-CT fragment were resolved in the cryo-EM map, we cannot identify if divergent residues in the interior surface of Tse5-Shell mediate protein-protein interactions with Tse5-CT.

Mutations on the outer surface of Tse5-Shell accumulate mainly around 10 regions, corresponding to the following residue ranges: 144-167, 230-235, 247-254, 362-364, 466-471, 676-680, 787-789, 873-895, 960-962, 1041-1062 (Supplementary Table 6). Regions 873-895, and 1041-1062 are particularly large and are within the *Predicted Helical Region 1* and *Helical Region 2*, respectively (Fig. 5a). Region 676-680 and residue 670 form a *Hydrophobic Patch* on the Tse5-Shell surface, which results in one side more amphipathic than the other (Fig. 5b; Supplementary Fig. 8).

Supplementary Tables

Supplementary Table 1 | Cryo-EM data collection processing and model refinement statistics.

**Data Collection**	**EMD 16778, PDB 8CP6**
Microscope	ThermoFisher Titan Krios
Magnification	130000x
Voltage (kV)	300
Camera	Falcon 4
Pixel size (Å/pixel)	0.921
Total electron dose ($e^-/Å^2$)	60
Exposure time (s)	10.84
Defocus range (μm)	-1-(-2)
Number of images	10244
**Processing**	
Number of selecteMicrographs	8930
Number of initial particles	4866119
Number of final particles	323,963
Final resolution (Å)	2.45
Symmetry	C1
Map sharpening B factor ($Å^2$)	82
**Structure Composition /Validation**	
No. of chains	3
Non-hydrogen atoms	8899
Protein residues	1129
Bond RMSD lengths (#>4 σ)	0.003 Å (0)
Bond RMSD angles (#>4 σ)	0.423° (0)
Molprobity score	1.28
Clashcore	5.51
Poor rotamers (%)	0.8
Ramachandran favored (%)	98.31
Ramachandran allowed (%)	1.69
Ramachandran outliers (%)	0
CC (mask)	0.84
CC (box)	0.76
CC (peaks)	0.72
CC (volume)	0.84

Supplementary Table 2 | Descriptive graph describing the type of protein-protein interactions between Tse5-NT and Tse5-Shell calculated using Mapiya online server [5].

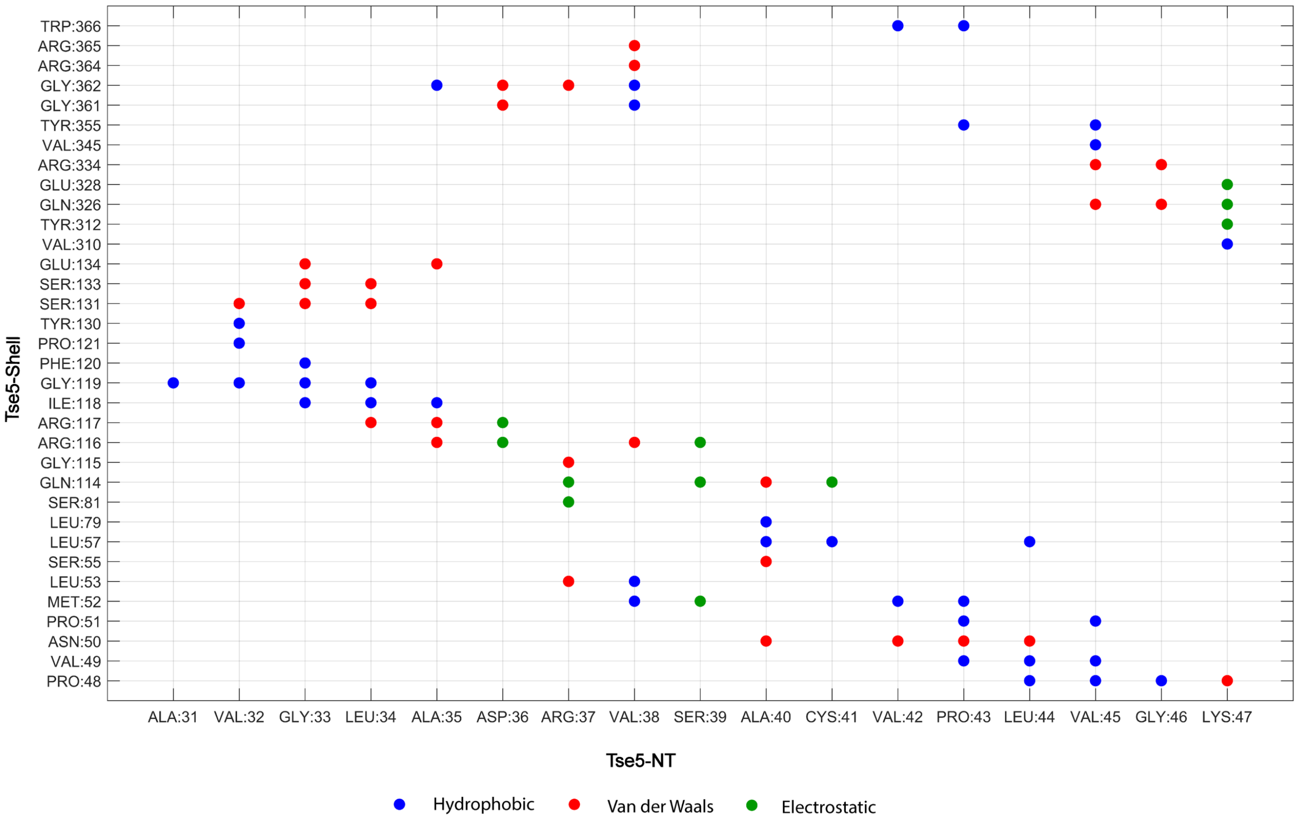

Supplementary Table 3 | Summary of Small-angle X-ray scattering data analysis for Tse5 and Tse5 variants.

Data-collection parameters	Tse5	Tse5-K47G-P48A	Tse5-D1141A	Tse5-ΔCT
Instrument	Beamline B21 (DLS)	Beamline B21 (DLS)	Beamline B21 (DLS)	Beamline B21 (DLS)
Wavelength (Å)	0.946	0.946	0.946	0.946
Strategy	SEC-SAXS	SEC-SAXS	SEC-SAXS	SEC-SAXS
q range ($Å^{-1}$) [b]	0.093-0.20	0.093-0.20	0.093-0.20	0.093-0.20
Exposure time (sec/frame)	3	3	3	3
Injected concentration ($mg{\cdot}mL^{-1}$)	2.5	2.5	2.5	2.5
Temperature (K)	288	288	288	288
**Structural parameters***				
$I(0)$ (cm^{-1}) [from P(r)]	0.020 ± 2.2e - 05	0.020 ± 1.8e - 05	0.021 ± 2.2e - 05	0.019 ± 2.4e - 05
R_g (Å) [from P(r)]	35.37 ± 0.04	35.49 ± 0.04	35.84 ± 0.04	35.27 ± 0.04
$I(0)$ (cm^{-1}) [from Guinier]	0.021 ± 3.6e - 05	0.021 ± 3.9e - 05	0.022 ± 4.3e - 05	0.019 ± 2.1e - 05
R_g (Å) [from Guinier]	35.72 ± 0.10	35.87 ± 0.10	36.18 ± 0.11	35.46 ± 0.11
d_{max} (Å)	107.5	107.5	107.5	107.5
Porod volume estimate ($Å^3$)	243,269	262,729	255,410	271,762
Dry volume calculated from sequence ($Å^3$)	180,017	179,899	179,9963	161,197
**Molecular-mass determination by Bayesian inference**				
MW Estimate (Da)	157,050	157,050	157,050	138,225
MW Probability (%)	61.87	47.17	54.99	42.22
Credibility (Da)	142,150 – 176,600	142,150 – 176,600	134,300 – 162,650	127,450 – 151,450
Credibility Interval Probability (%)	98.43	91.99	93.69	97.88
Calculated monomeric from sequence (Da)	148,776	148,732	148,679	133,223
**Software employed**				
Primary data reduction	GDA	GDA	GDA	GDA
Data processing	ScÅtter IV/ PRIMUS	ScÅtter IV / PRIMUS	ScÅtter IV / PRIMUS	ScÅtter IV / PRIMUS

[b] q-range used for calculation of P(r) function

Supplementary Table 4 | Strains and Plasmids used in this study.

Strain	Relevant characteristics	Use	Origin
Escherichia coli			
**Lemo21**	BL21(DE3) strain with an extra plasmid harbouring the gene encoding T7 lysozyme, an inhibitor of the T7 RNAP, under control of the well-titratable rhamnose promoter	Heterologous expression of Tse5 for purification of Tse5-CT	[2]
P. aeruginosa			
**D*retS***	*P. aeruginosa* PAO1 strain lacking the regulator *retS*	Donor strain for intraspecific bacterial competition assays	[3]
**D*retS* D*tse5* D*tsi5* pPSV35-CV**	*P. aeruginosa* PAO1 strain lacking the regulator *retS* and the Tse5 effector/immunity pair, harbouring a plasmid that confers gentamicin resistance	Recipient strain for intraspecific bacterial competition assays	[4]
**D*retS* Tse5_D1141A**	*P. aeruginosa* PAO1 strain lacking the regulator *retS*, in which the native Tse5 allele has been replaced with one bearing the D1141A mutation	Donor strain for intraspecific bacterial competition assays	This study
**D*retS* Tse5_D1164A**	*P. aeruginosa* PAO1 strain lacking the regulator *retS*, in which the native Tse5 allele has been replaced with	Donor strain for intraspecific bacterial competition assays	This study

	one bearing the D1164A mutation		
**D*retS* Tse5_K47G_P48A**	*P. aeruginosa* PAO1 strain lacking the regulator *retS*, in which the native Tse5 allele has been replaced with one bearing the K47G-P48A mutation	Donor strain for intraspecific bacterial competition assays	This study
**Plasmids**	**Relevant characteristics**	**Use**	**Origin**
**pET29a(+)::*9xhis-Tse5***	Plasmid harbouring a construct based on tse5 and coding for a 9xHis tag and a tobacco etch virus protease cleavage site at the 5' end.	Heterologous expression of Tse5 in *E. coli* Lemo21 cells for purification of Tse5	This work
**pET29a(+)::*D1141A***	Plasmid derived from pET29a(+)::*9xhis-Tse5* coding for a D1141A point mutation.	Test the activity of the putative aspartyl protease motif DPXGL-(18)-DPXGL	This work
**pET29a(+)::*D1164A***	Plasmid derived from pET29a(+)::*9xhis-Tse5* coding for a D1164A point mutation.	Test the activity of the putative aspartyl protease motif DPXGL-(18)-DPXGL	This work
**pET29a(+)::*K47G-P48A***	Plasmid derived from pET29a(+)::*9xhis-Tse5* coding for a K47GP48 point mutations.	Test the relevance of the proteolysis of the N-terminal domain	This work
**pET29a(+)::Tse5-ΔCT**	Plasmid derived from pET29a(+)::*9xhis-Tse5* coding the deletion of Tse5_CT.	Test the activity of Tse5 in the absence toxic Tse5_CT domain	This work

Supplementary Table 5 | Residues lining the interior of the Tse5-Shell that diverge from the consensus sequence found in Tse5 homologues containing C-terminal toxic fragments of putative/known enzymatic functions.

RES. # Tse5	RES. Tse5	CONSENSUS RES.	RESIDUE # Tse5	RES. Tse5	CONSENSUS RES.
**67**	A	P	**543**	A	P
**70**	D	L	**573**	W	Y
**71**	T	P	**585**	A	P
**99**	M	gap	**594**	H	Y
**154**	R	gap	**597**	A	E
**195**	G	D	**619**	D	G
**304**	E	D/N	**640**	D	G
**328**	E	G	**679**	E	gap
**362**	G	gap	**708**	N	gap
**371**	R	D	**709**	L	gap
**402**	R	Y	**737**	S	R
**423**	V	Y	**773**	H	G
**456**	G	D/N	**805**	S	Q
**472**	D	G	**977**	S	G
**506**	W	T	**1158**	A	gap
**520**	S	T	**1166**	T	L

Residues colour-coded following the Lesk scheme
(Introduction to Bioinformatics)

Small nonpolar	G, A, S, T	**Orange**
Hydrophobic	C, V, I, L, P, F, Y, M, W	**Green**
Polar	N, Q, H	**Magenta**
Negatively charged	D, E	**Red**
Positively charged	K, R	**Blue**

Supplementary Table 6 | Residues lining the exterior of the Tse5-Shell that diverge from the consensus sequence found in Tse5 homologues containing C-terminal toxic fragments of putative/known enzymatic functions.

RESIDUE # Tse5	RESIDUE Tse5	CONSENSUS RES.	RESIDUE # Tse5	RESIDUE Tse5	CONSENSUS RES.
53	L	T	676	A	deletion
144	S	deletion	677	L	deletion
145	G	deletion	678	V	deletion
146	G	deletion	679	E	deletion
147	E	X	680	R	deletion
148	A	deletion	684	L	D
149	Q	deletion	708	N	deletion
150	A	X	720	W	deletion
151	W	Y	750	Y	D
152	R	deletion	773	H	G
153	G	deletion	779	D	deletion
154	R	deletion	780	A	deletion
155	W	deletion	781	R	deletion
156	A	deletion	787	D	deletion
157	A	deletion	788	A	deletion
158	V	deletion	789	L	deletion
159	P	deletion	805	S	Q
160	A	deletion	836	P	G
161	E	deletion	851	S	deletion
162	L	deletion	873	V	deletion
163	Q	deletion	874	G	deletion
164	T	deletion	875	E	deletion
165	Q	deletion	876	H	deletion
166	E	deletion	877	A	deletion
167	G	deletion	878	R	deletion
230	C	deletion	879	E	deletion
231	E	deletion	880	D	deletion
232	P	deletion	881	A	deletion
233	S	deletion	882	R	deletion
234	E	deletion	883	Q	deletion
235	G	deletion	884	A	X
247	A	deletion	885	F	X
248	S	deletion	886	A	X
249	H	deletion	887	E	deletion
250	D	deletion	888	N	deletion
251	G	deletion	889	E	deletion

252	P	deletion
253	P	deletion
254	P	deletion
266	W	deletion
272	F	Y
291	V	deletion
294	W	Y
299	L	X
304	E	D/N
306	G	N
314	W	Y
318	A	D
328	E	G
358	A	N
362	G	deletion
363	Q	deletion
364	R	deletion
371	R	D
402	R	Y
416	K	G
423	V	Y
438	Q	G
456	G	D/N
466	E	D
467	D	X
468	P	deletion
469	R	deletion
470	L	X
471	A	X
490	N	D
520	S	T
543	A	P
573	W	Y
585	A	P
594	H	Y
670	A	deletion

890	L	deletion
891	Y	deletion
892	R	deletion
893	S	deletion
894	G	deletion
895	F	deletion
948	Y	W
957	L	V
960	D	deletion
961	Y	deletion
962	A	P
966	R	deletion
967	L	deletion
993	V	T
1041	L	deletion
1042	R	deletion
1043	Q	deletion
1044	A	deletion
1045	F	deletion
1046	A	deletion
1047	A	deletion
1048	E	deletion
1049	G	deletion
1050	Q	deletion
1051	P	deletion
1052	L	deletion
1053	P	deletion
1054	A	deletion
1055	Q	X
1056	C	X
1057	V	X
1058	P	X
1059	A	deletion
1060	L	deletion
1061	G	deletion
1062	E	deletion

Residues colour-coded following the Lesk scheme (Introduction to Bioinformatics)

Small nonpolar	G, A, S, T	**Orange**
Hydrophobic	C, V, I, L, P, F, Y, M, W	**Green**
Polar	N, Q, H	**Magenta**
Negatively charged	D, E	**Red**
Positively charged	K, R	**Blue**

Supplementary Figures

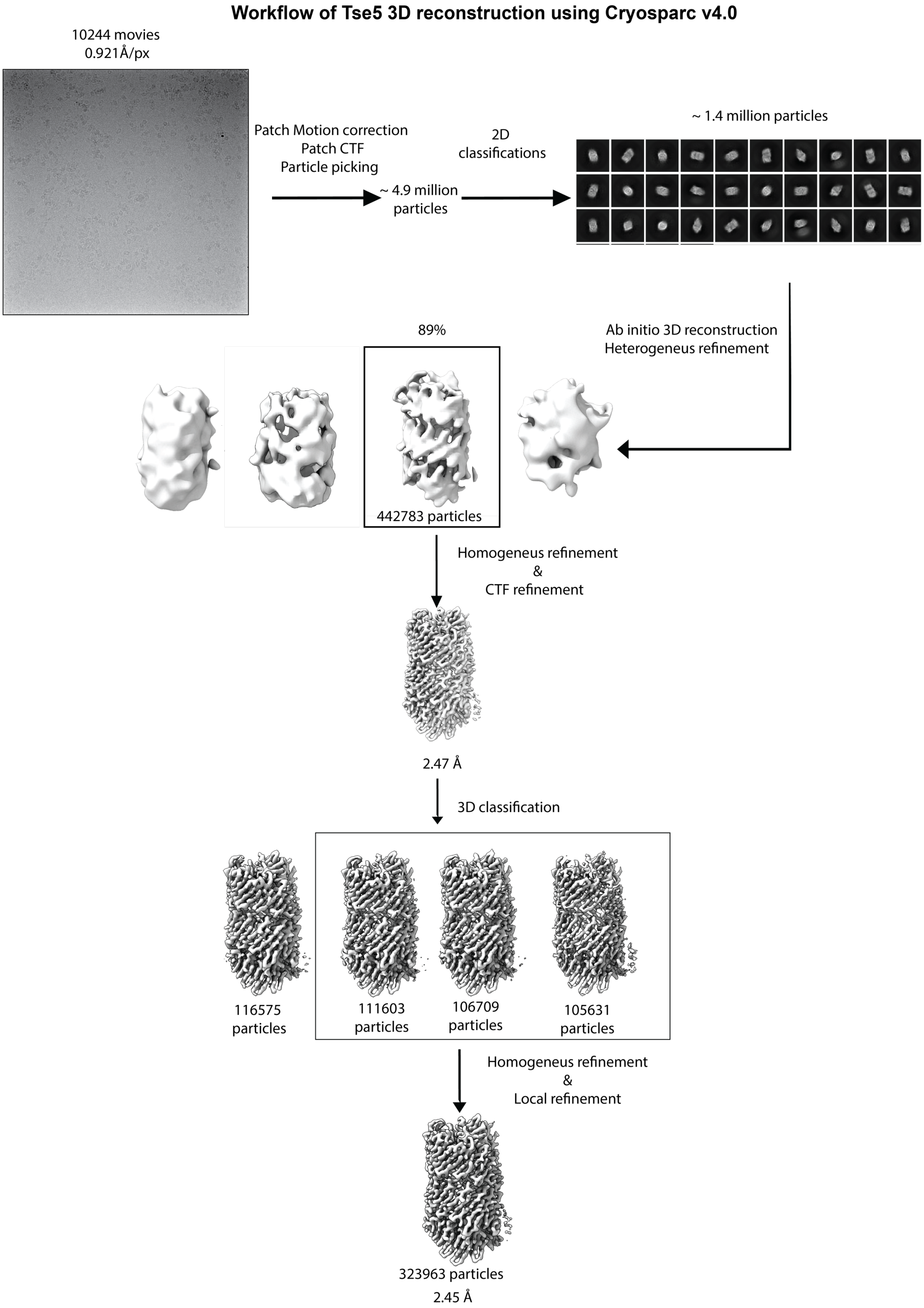

Supplementary Figure 1 | Workflow for cryo-EM structure determination.
Scheme of the cryo-EM processing for the *P. aeruginosa* Tse5 effector, from data collection to the final map at 2.45 Å, as internally calculated by cryoSPARC.

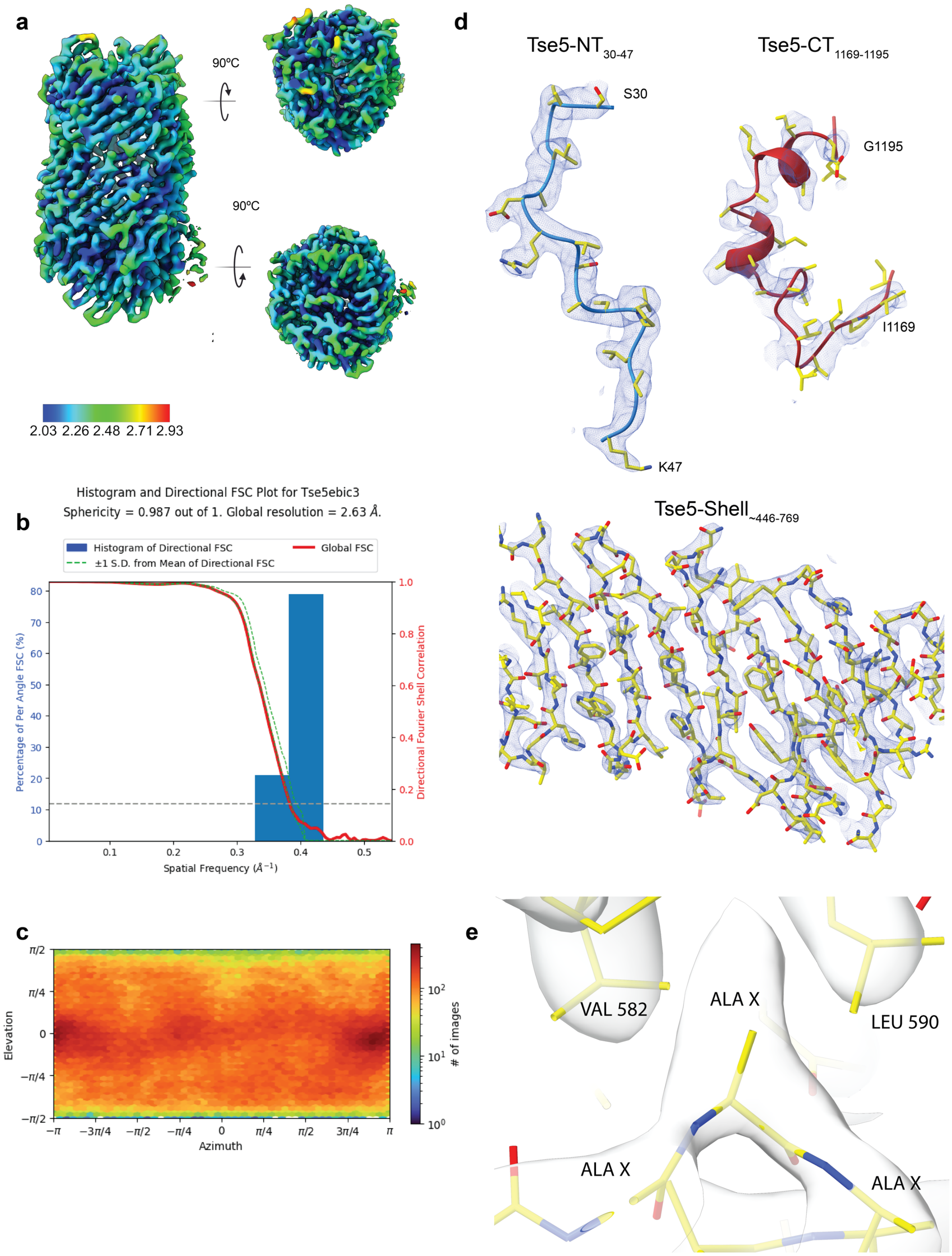

Supplementary Figure 2 | Cryo-EM Structural determination of Tse5.

a. Side view and top views of the Tse5 cryo-EM map in surface representation, local resolution is plotted onto the surface. **b.** Fourier shell correlation plot calculated from two half-maps and resolution anisotropy, obtained by 3DFSC online server tool. Sphericity indicates the anisotropy degree of the 3D reconstruction and the histogram indicates the percentage of voxels with a certain resolution. Resolution estimation is reported at the gold standard cutoff of 0.143 Fourier Shell Correlation (FSC)

of the final reconstruction. **c.** Angular distribution plot of the particles calculated by Cryosparc. The map crosses the 0.143 FSC threshold at 2.63 Å while the FSC curve calculated with cryoSPARC or *phenix.mtriage* [6] crosses at 2.45 Å. **d.** Representative fragments of the Tse5 cryo-EM map in mesh layout, with respective modelled residues shown as a stick and cartoon representation. **e.** Part of the Tse5-CT unassigned map shows a residue inserted in between two hydrophobic residues of the Tse5-Shell's inner cavity.

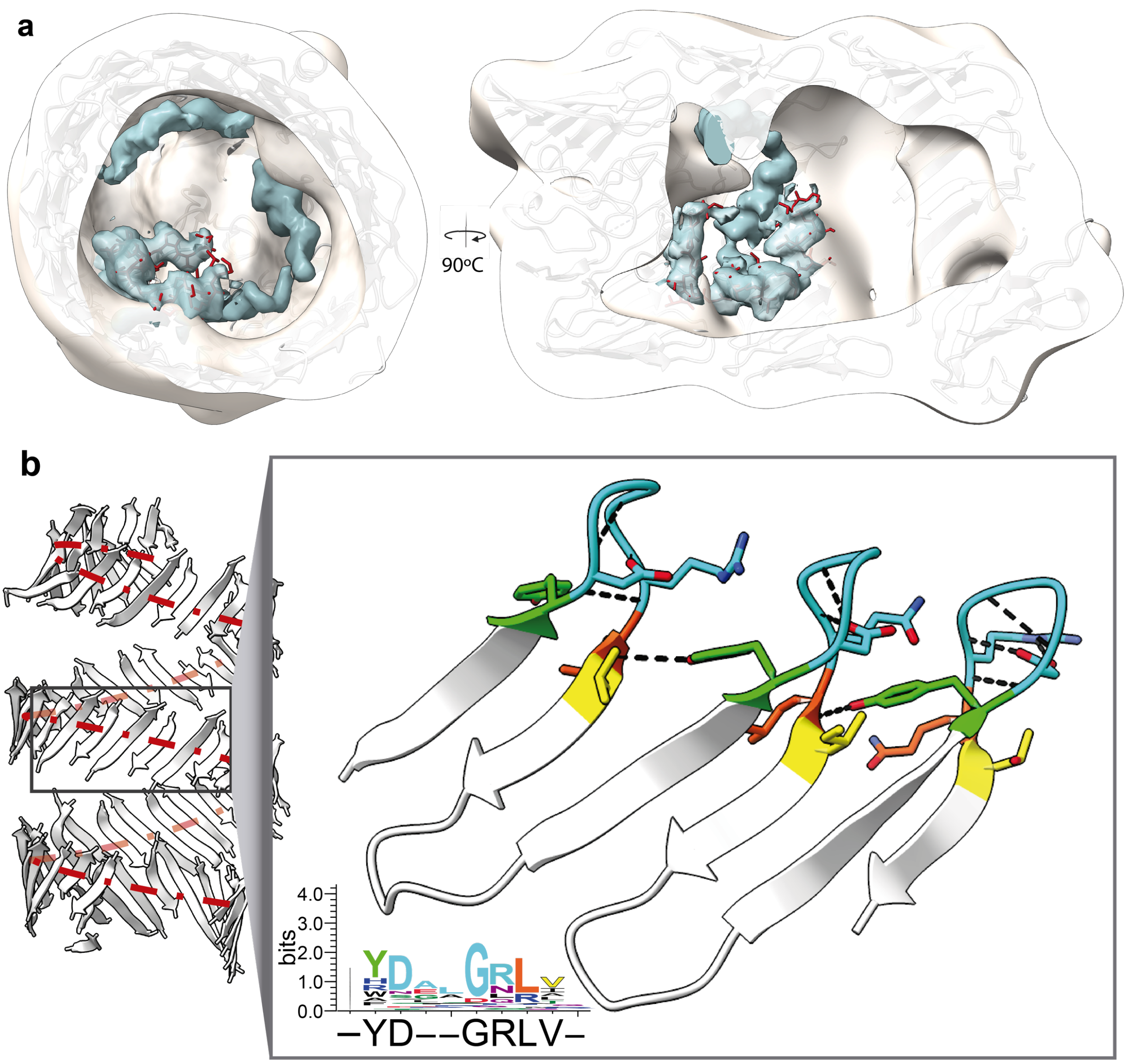

Supplementary Figure 3 | Tse5-Shell encapsulates the pore-forming Tse5-CT toxin

a. Two views rotated 90° showing the Tse5-Shell's cavity. Only the first 27 residues of Tse5-CT (1169-1195) could be resolved inside the cavity, although some extra density remains unassigned (shown as a solid surface). The size of this cavity (~29,000 Å^3) is sufficient to accommodate the ~16 kDa Tse5-CT toxin. **b.** The Tse5-Shell is formed by a β-sheet assembled from 40 repeating β-hairpins with the consensus sequence YDXXGRLV. The DXXGR motif that creates the turn of each β-hairpin is shown in blue. The hydrogen bonds within the turn are shown as dotted lines. The hydrogen bond between the tyrosine residues in the YD motifs and the leucine residue/position of the preceding YD-repeat is also shown with dotted lines.

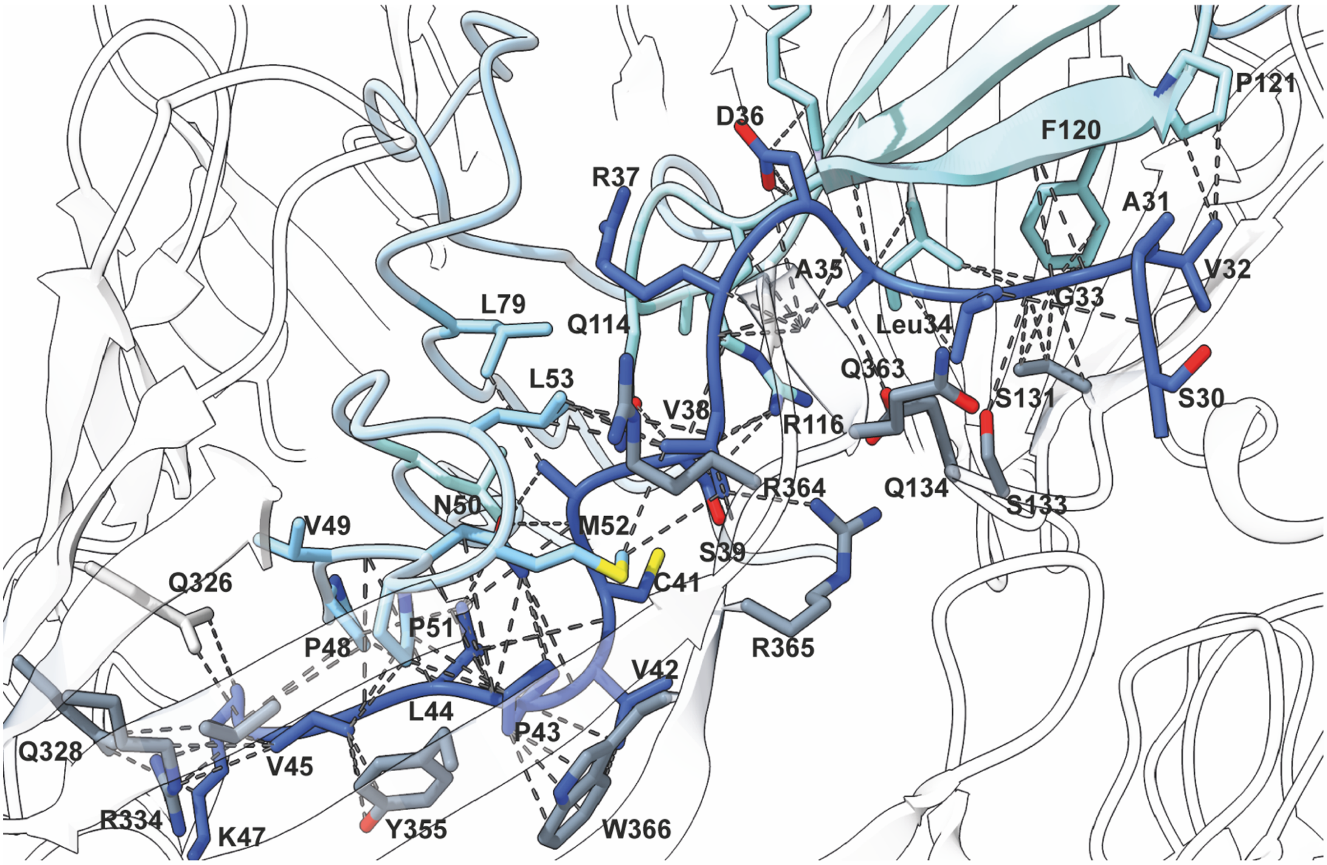

Supplementary Figure 4 | Residues Ser32-Lys47 anchor Tse5-NT fragment to Tse5-Rhs fragment through protein-protein interactions.

Tse5-NT and Tse5-Shell's N-terminal plug are coloured in blue and light blue, respectively. Residues involved in the interactions can be visualised in stick representation. The remaining Tse5-Shell is depicted in white (cartoon representation), and residues interacting with Tse5-NT are marked in grey (stick representation). The interactions are shown in dotted lines, and black labels indicate the type and residue number.

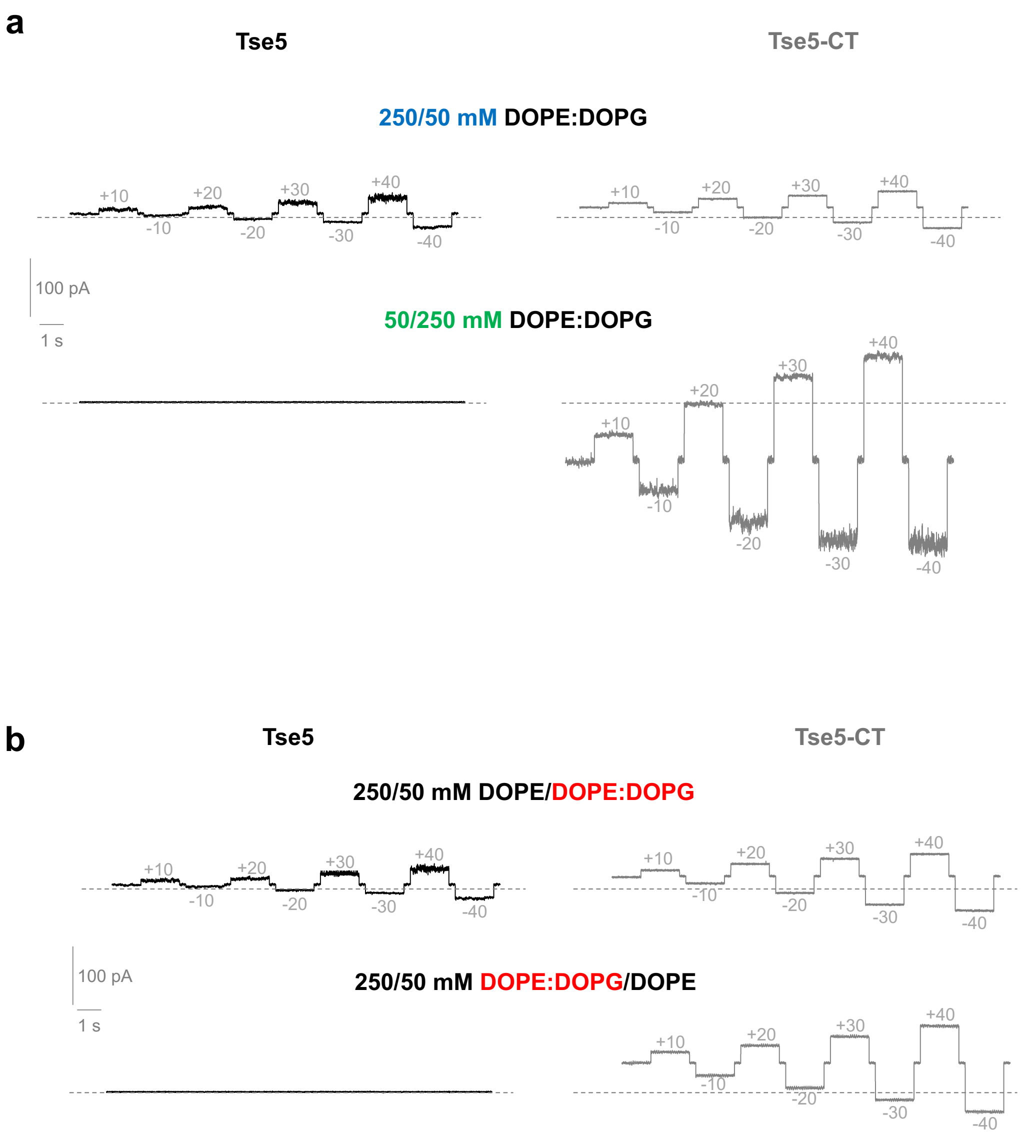

Supplementary Figure 5 | Tse5 is sensible to the membrane potential directionality.

a. Representative current traces induced by Tse5 (n = 15) or Tse5-CT (n = 9) were obtained in a 250/50 mM KCl gradient using a negatively charged DOPE:DOPG (67:33 w/w) lipid mixture to form the membrane. When the gradient is set to 50/250 mM, Tse5 cannot form ion-permeable pores (n = 9), contrary to Tse5-CT (n = 8). Number of replicates (n) with similar results are indicated for each representative current traces shown in the figure **b.** Traces of Tse5 (n = 9) and Tse5-CT (n = 17) recorded using a neutral lipid (DOPE) on the *cis* side and a charged lipid mixture (DOPE:DOPG 67:33 w/w) on the *trans* side (upper panel). The lower panel shows a representative trace in 250/50 mM KCl gradient when a charged lipid mixture (DOPE/DOPG 67:33 w/w) was used on the side of protein addition (*cis* side), and a neutral DOPE lipid on the opposite side. Tse5 could not generate pores (n = 7) when the charged lipid was present on the *cis* side, contrary to Tse5-CT (n = 14). The applied voltages in millivolts are shown in light grey. All records were digitally filtered with 500 Hz using a low-pass 8-pole Bessel filter to enhance visualisation. Dashed lines indicate a zero current level.

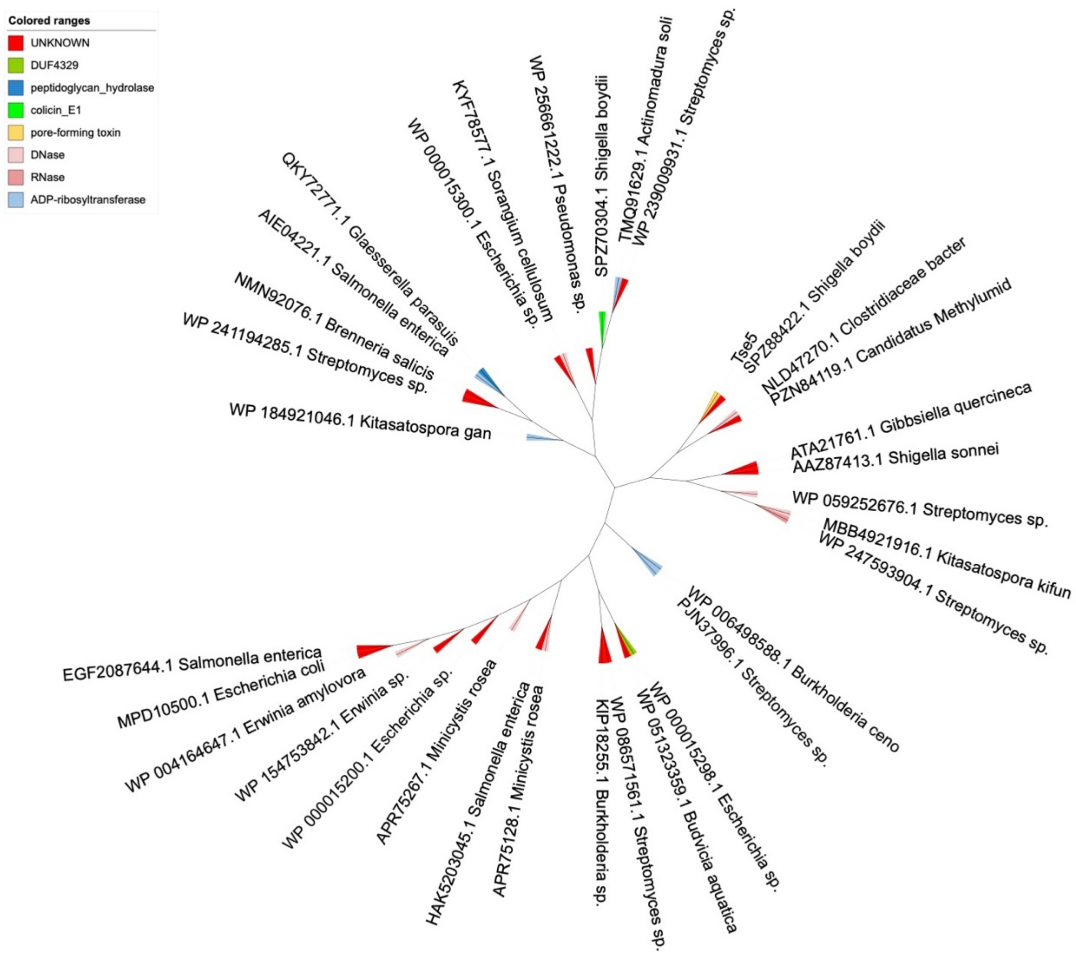

Supplementary Figure 6 | Phylogenetic analysis of C-terminal fragments found in Tse5 homologues.

Phylogenetic analysis of C-terminal fragments found in Tse5 homologues identified by Foldseek [1]. Putative functions of C-terminal toxins are indicated in the top-left corner. Phylogenetic tree display was generated with iTOL [7].

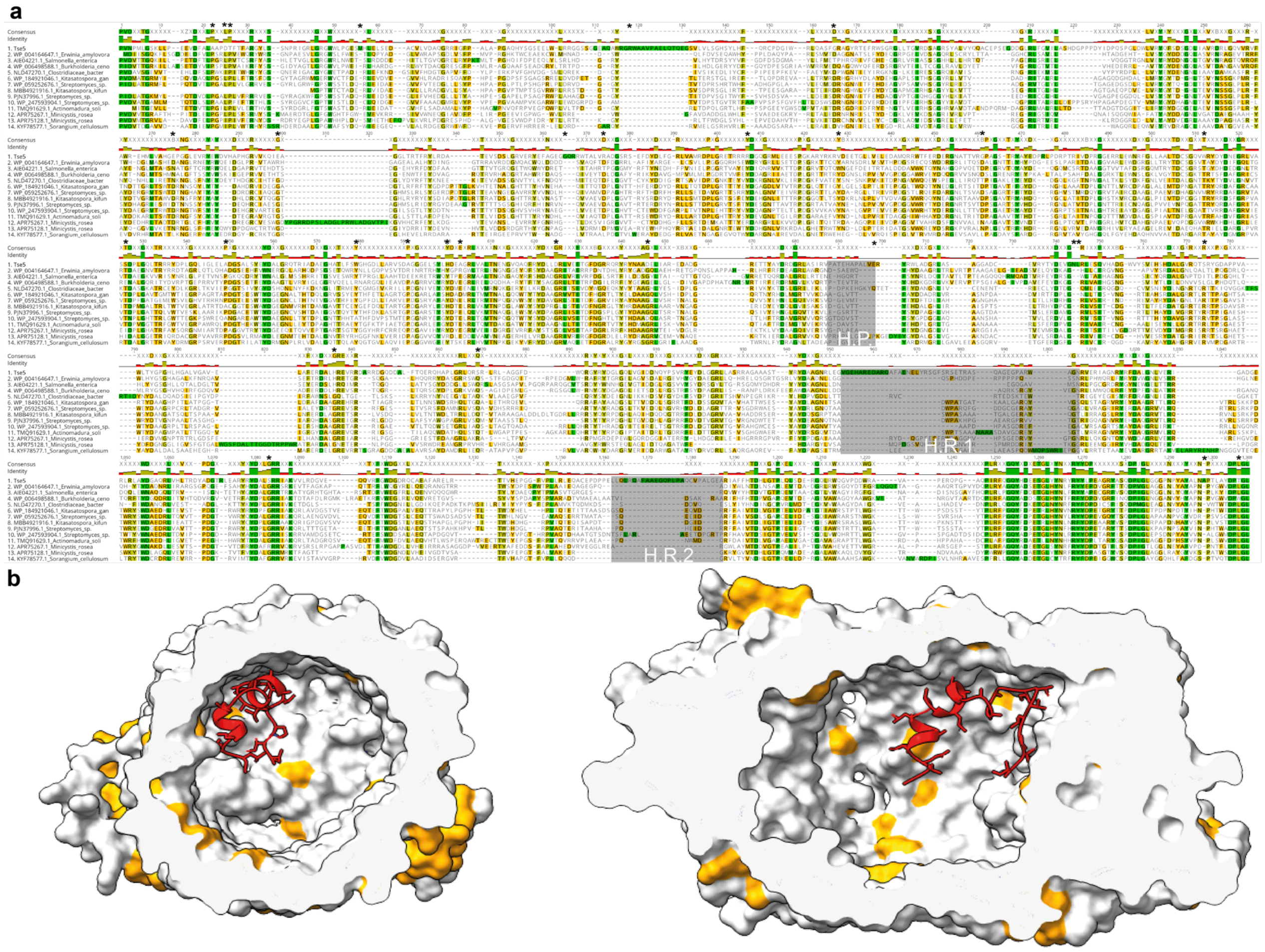

Supplementary Figure 7 | Alignment of Tse5 with homologues containing C-terminal toxic fragments of putative/known enzymatic functions

a. The Hydrophobic Patch (H.P.) and Helical Regions 1 and 2 (H.R.1 and H.R.2.) in Tse5, as well as residues lining the interior of the cocoon that diverge from the consensus sequence, are indicated with grey boxes or a star mark, respectively. The Consensus threshold was set to 65% (a residue is conserved if at least it is present in 65% of aligned sequences). Residues are colour-coded following the following similarity code: 100% similar (Green); 80 to 100% similar (olive green); 60 to 80% similar (yellow); less than 60% similar (white). The consensus and identity of the aligned sequences are shown on top. **b.** The surface of Tse5 residues lining the interior/exterior of the cocoon that diverge from the consensus sequence are coloured in yellow. The Helical Region 1 (residues 873-895) was modelled to map sequence divergence.

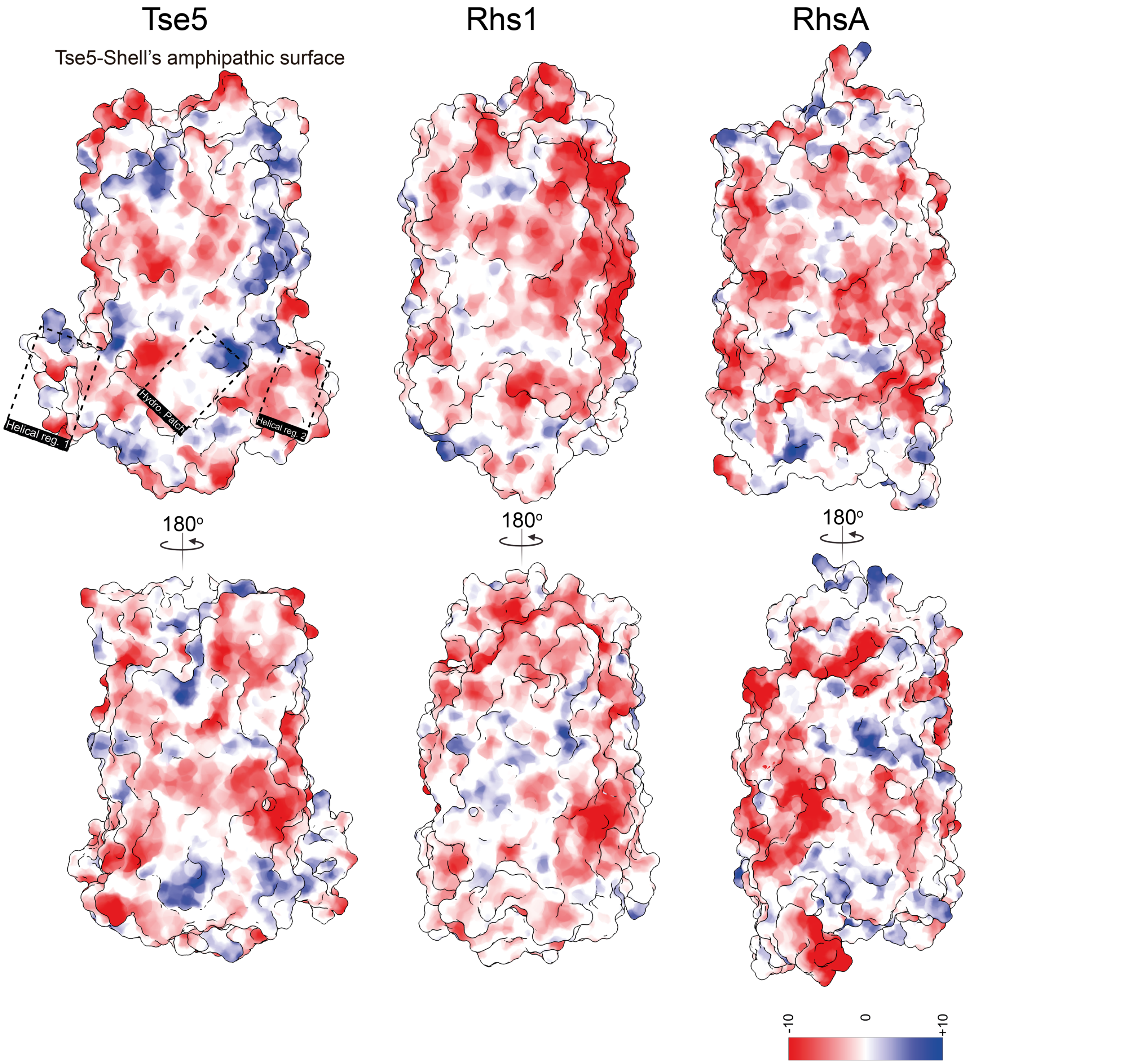

Supplementary Figure 8 | Electrostatic surface potential of Tse5, Rhs1 and RhsA.

Figure showing two views of Tse5, Rhs1, and RhsA rotated 180º along the long axis. The colour key bellow indicates the electrostatic surface potential values plotted onto the surface (−10 to +10 kcal/(mol·e⁻) at 298 K). The Coulombic electrostatic potential was calculated by ChimeraX.

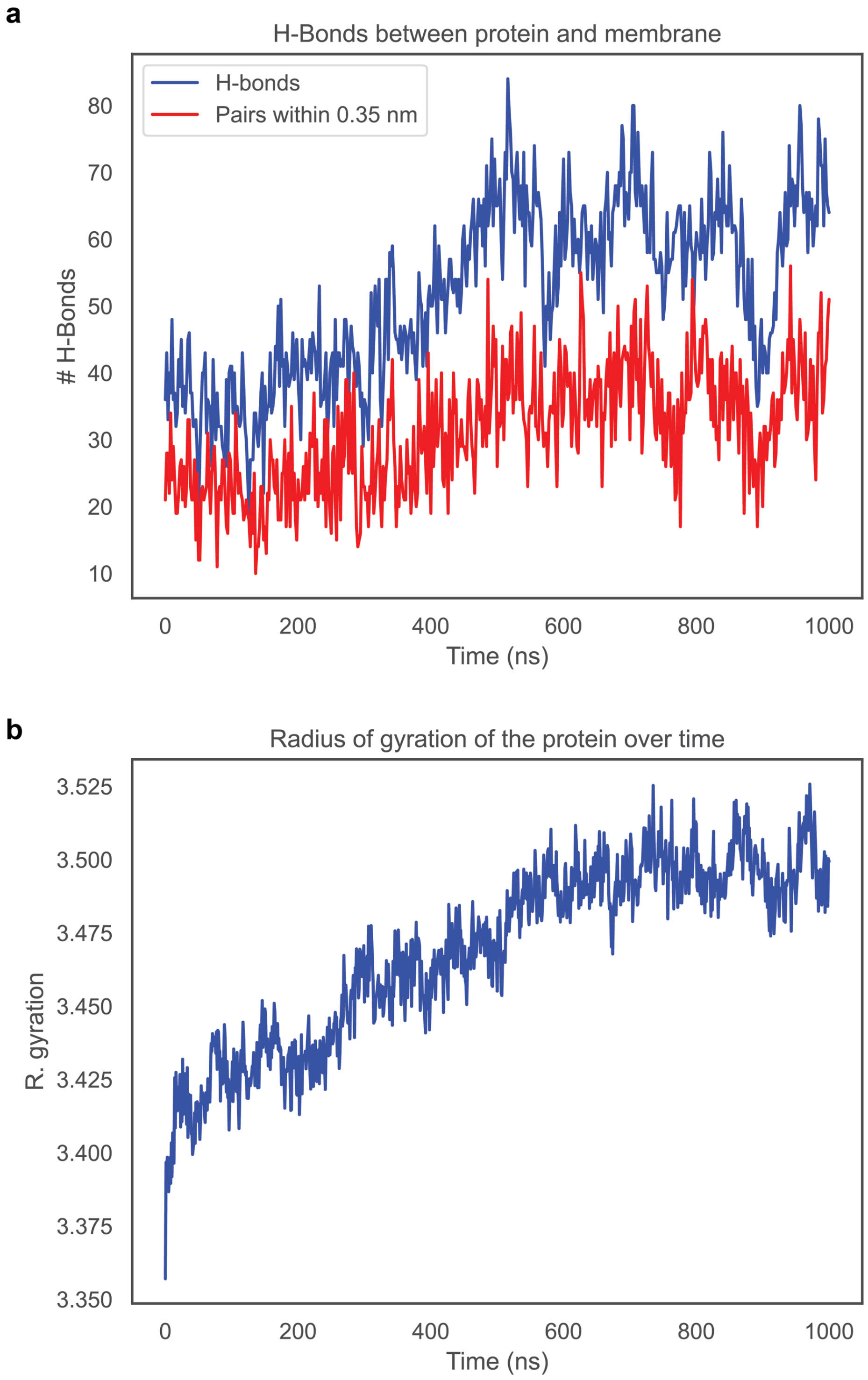

Supplementary Figure 9 | Protein-membrane interactions and protein stability during MD simulation

a. Plot showing h-bonds (blue), and atom pairs closer than 3.5 Å (red) between the protein and the membrane in the MD trajectory over the 1 µs MD simulation. Both measures increase with time until a putative fluctuating stability is reached. **b.** Plot showing the radius of gyration change of Tse5 during the 1 µs MD simulation. Although the visual increase of the radius of gyration of the protein, the change in time is residual, indicating the overall stability of the protein throughout the the 1 µs MD simulation

MMGSSHHHHHHHHHHSSGENLYFQGGSMSGLPVSHVGEKVSGGVISTGSPTVHVGSSAVGLA
DRVSACVPLVGKPVNPMLGSKLLPEEVDFALAAPDTFTFARGYLSSNPRIGRLGRGWWLPG
ESMHLELSEDACVLVDAQGRRIGFPALAPGAQHYSGSEELWLRRGGSSGGEAQAWRGRWAA
VPAELQTQEGSVLVLSGHSYLHFQRCPDGIWRLQASFGRAGYRTEFRWSGRGLLTGVRDSA
GRSYALVYQQACEPSEGDDGLRLFGVILASHDGPPPDYIDPQSPGLDWLVRYQFSDSGDLI
AVRDRLGQVVRVFAWREHMLVAHGEPGGLEVRYEWDVHAPHGRVVKQIEAGGLTRTFRYLR
DATEVSDSLGRVERYEFAGEGGQRRWTALVRADGSRSEFDYDLFGRLVAMRDPLGRETRRR
RDGQGRMLEEESPGKARYRKRVDEETGLLVELEDAMQRRWTFERDERGNATTVRGPAGSTR
YAYEDPRLPDRPTRIVDPRGGERRLEWNRFGLLAALTDCSGQVWRYDYDNEGRLVASSDPL
GQLTRRRYDPLGQLIGLELADGSALSYEYDALGRQTRIADAEGHATLFSWGHGDLLARVSD
AGGGELSYLHDEAGRLVALTNENGVQAQFRYDLLDRLVEETGFDGRRQRYRYNAADELIAR
EDADGRETTYAYDRDGRLASIRVPATEHAPALVERYRWLADGRLASAGGADCEVRYTYDEV
GNLRLESQVHADGWVYSVEHSHDALGVRQTSRYGDAPPVAWLTYGPGHLHGALVGAVELAF
ERDALHREVRRDARRDGQDDALFTQERQHAPLGRLQRSRLRLAGGFDWQRGYRYDGLGQLV
GIDDNQYPSVRYEYDLGGRLLASRRAGAAASTYRYDAAGNRLEGVGEHAREDARQAFAENE
LYRSGFSRSETRASQAGEGPARWAGNRVERIAGNRYRFDALGNLVERIGADGERLRLAYDG
AQRLVHLTRDYADGTRLEARYRYDALSRRIAKVVLRDGVEQQVRFGWDGDRQCAEAFAREL
RTTVHEPGGFVPLLRLEQACEPDPPELLQLRQAFAAEGQPLPAQCVPALGEARIAFFHTDH
LGTPLQLSDERGQLRWQGVPDDWRAVAPERQPGAQPIRFQGQYHDEESGLYYNRYRYYLPE
AGRYASQDPLGLGGGPNPYAYALNAPTLAYDPTGLIIPLVVIGAFAARAAIGAALGAGIEL
GMQTGKQVLGQMKDNWDSDRDLTDIKWKCIDINWKHVGASAAIGTVAPGMLSTGKTVVQSA
KAIRTLSGQAANTANRAAKLAARKAAHADTIKKAVATQAAWQTGKQIVKCPLKDEEEECPP
Q

His-tag

Tse5-NT **Tse5-CT**

NT-plug **CT-plug**

Supplementary Figure 10 | Tse5 sequence derived for structural and biophysical studies.

His-tag with the tobacco etch virus protease cleavage site is indicated with grey shading. Residues corresponding to the Tse5 N-terminal and the C-terminal fragments are coloured in dark blue and red, respectively. Residues corresponding to the N-terminal and C-terminal plugs are coloured in light blue and salmon, respectively.

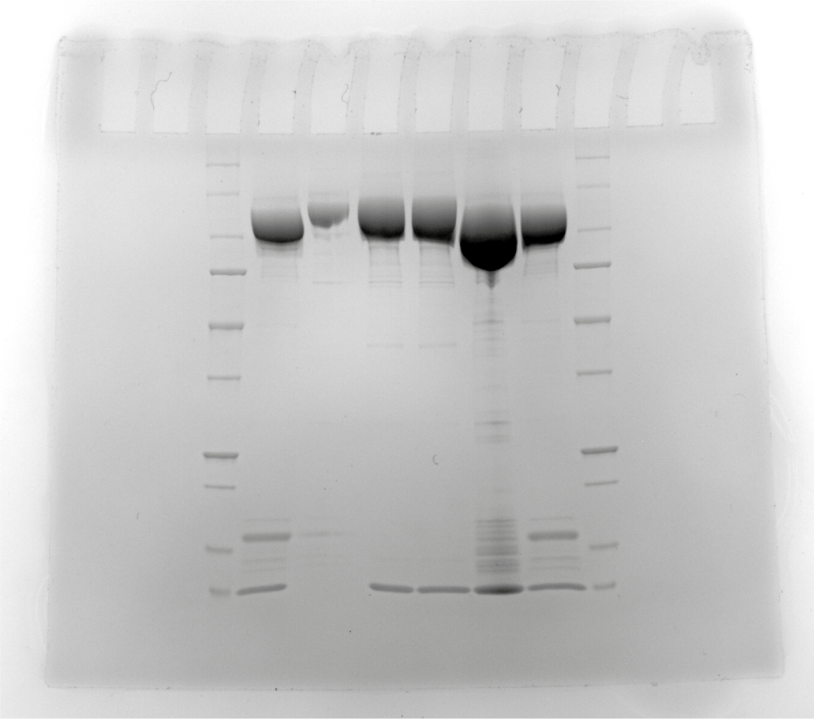

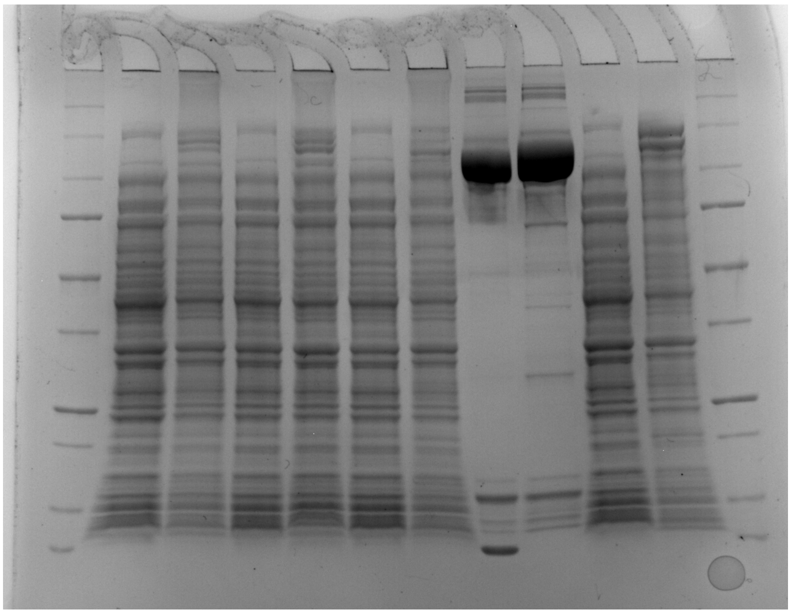

Supplementary Figure 11 | Uncropped and unedited SDS-gels

Supplementary Note 2: LC-ESI-MS report for Tse5

f · g · c · z
functional genomics center zurich

Dr. Serge Chesnov
Protein Analysis Group
Functional Genomics Center Zurich
Y 32 H 92B
Winterthurerstrasse 190
CH-8057 Zürich

Tel. +41 44 635 39 50
serge.chesnov@fgcz.uzh.ch

Date:	02.07.2020
Customer:	D. Albesa-Jove
Order/Samples:	13382
Analysis ID:	20200702

Sample preparation
- ☐ Reduction/alkylation
- ☐ Proteolytic digestion
- ☒ ZipTip
- ☐ Precipitation
- ☐ Prosorb
- ☐ Electroblotting
- ☐ Other

Separation
- ☐ HPLC
- ☐ Gel electrophoresis

Mass determination
- ☒ Electrospray
- ☒ MALDI
- ☒ LC/ESI
- ☐ LC/MALDI

Protein/peptide identification/characterization
- ☐ PMF MALDI
- ☐ PMF LC/MS
- ☐ MALDI/MS/MS
- ☐ ESI/MS/MS
- ☐ LC/MALDI/MS/MS
- ☐ LC/ESI/MS/MS

Amino acid analysis
- ☐ Composition
- ☐ Concentration

Sequence/structural analysis
- ☐ Edman degradation
- ☐ ESI/MS/MS
- ☐ MALDI/MS/MS

Comments

Due to somewhat enhanced sample heterogeneity (proven by direct-infusion ESI-MS and MALDI-MS), the ample was analysed in an LC-ESI-MS approach.
The sample was 3 fold diluted with 1% TFA and transferred to an autosampler vial for LC/MS. 1ul resp. 5 ul of sample were injected into an ACQUITY UPLC@ BioResolve-RP-mAb 2.7µ 2.1x150 450 A (Waters, USA) column. For separation and elution on an

1/2

Acquity UPLC station, a gradient buffer A (0.1% FA in water)/ buffer B (0.1% FA in AN) at a flow rate 200ul/min at 50^0 C over 25 min was applied.

Time	Flow (ml/min)	%A	%B	Curve
Initial	0.2	85	15	Initial
3.0	0.2	45	55	6
20.3	0.2	20	80	6
21.3	0.2	85	15	6
25	0.2	85	15	6

The analysis was performed on a Synapt G2 mass spectrometer directly coupled to the UPLC station.

Mass spectra were acquired in the positive-ion mode by scanning an m/z range from 100 to 4000 da with a scan duration of 1 s and an interscan delay of 0.1s. The spray voltage was set to 3 kV, the cone voltage to 50V, and source temperature 80 °C. The data were recorded with the MassLynx 4.2 Software (both Waters,UK). The recorded m/z data were then deconvoluted for single peaks into mass spectra by applying the maximum entropy algorithm MaxEnt1 (MaxLynx) with a resolution of the output mass 1 Da/channel and Uniform Gaussian Damage Model at the half height of 1Da.

The sample is quite heterogeneous; molecular weights of all major species could be computed. It has to be pointed out that, due to low MS performance, more material (5 ul) was loaded than the column capacity allows. For better data presentation, the chromatogram for 1ul injected is depicted. Based on the values of the detected intact masses and some experience, the cleavage sites of the detected species could be assigned.

1. 4.06 min: 7’442 da: Met1-Lys73 (**Lys73 -->Lys47 as per ORF**) + its acetylated form (7’484 da)
2. 4.58 min: 125’815 da: Leu216-Gln1343 (**Leu190-Gln1317 as per ORF**)(lower accuracy, due to poor MS respond and high mass)
3. 5.53 min: 15’570 da: Ile1195-Gln1343 (**Ile1169-Gln1317 as per ORF**)

2/2

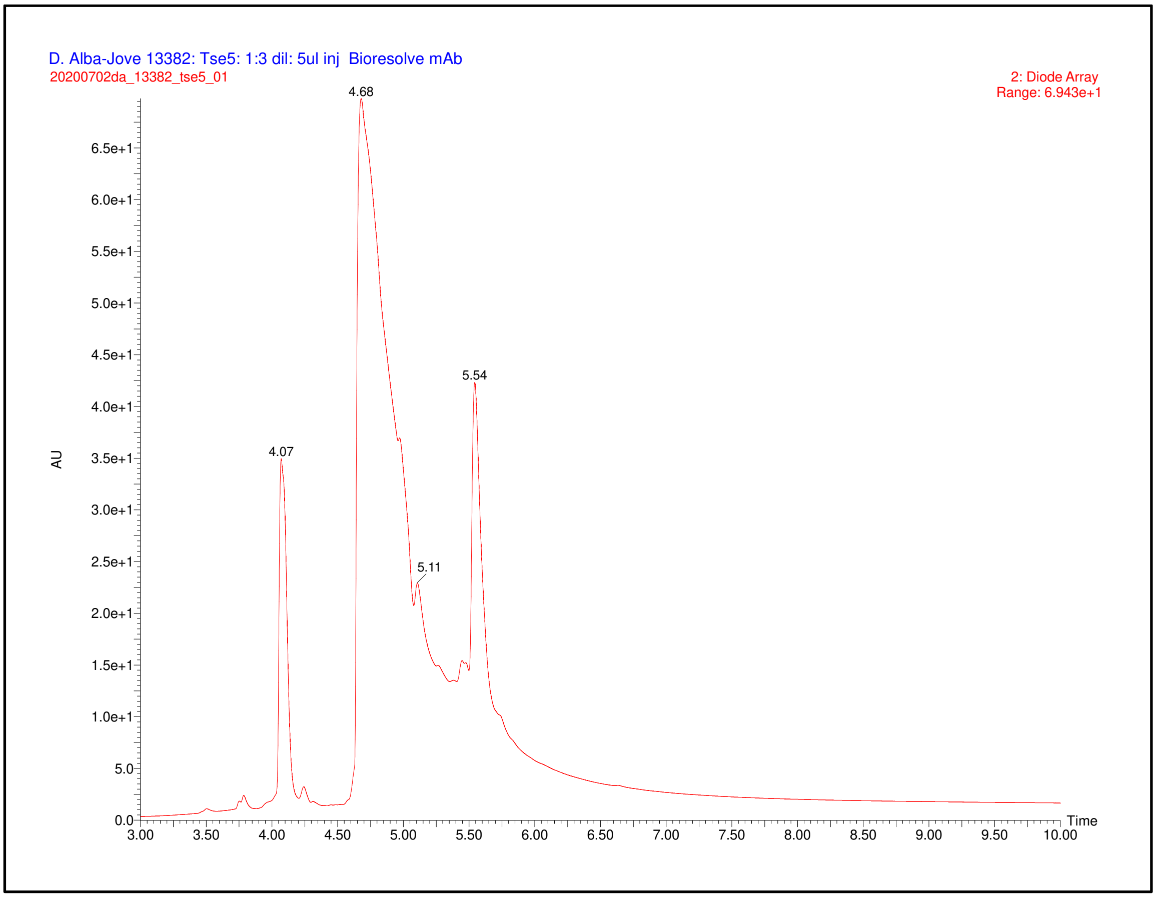
D. Alba-Jove 13382: Tse5: 1:3 dil: 5ul inj Bioresolve mAb
20200702da_13382_tse5_01
2: Diode Array
Range: 6.943e+1
4.68
5.54
4.07
5.11
AU
Time
6.5e+1
6.0e+1
5.5e+1
5.0e+1
4.5e+1
4.0e+1
3.5e+1
3.0e+1
2.5e+1
2.0e+1
1.5e+1
1.0e+1
5.0
0.0
3.00 3.50 4.00 4.50 5.00 5.50 6.00 6.50 7.00 7.50 8.00 8.50 9.00 9.50 10.00

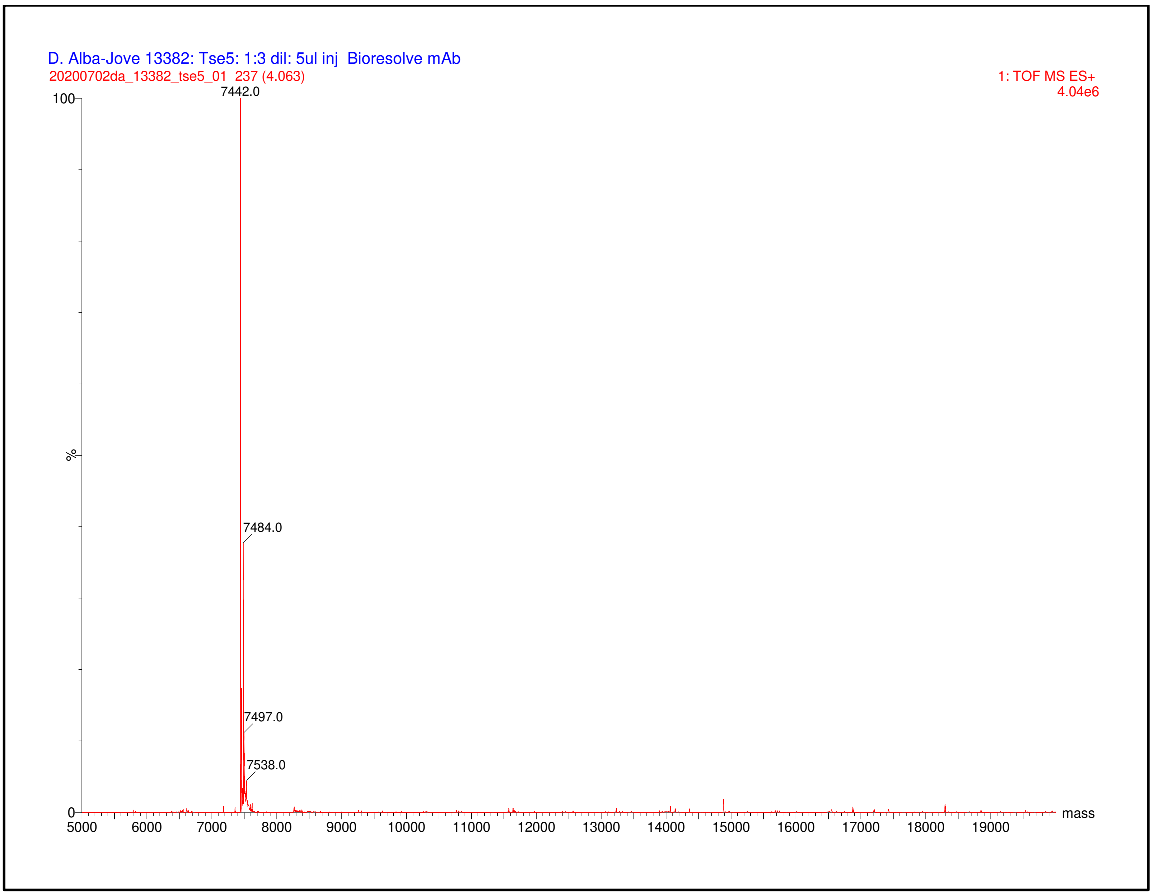
D. Alba-Jove 13382: Tse5: 1:3 dil: 5ul inj Bioresolve mAb
20200702da_13382_tse5_01 237 (4.063)
1: TOF MS ES+
4.04e6
7442.0
7484.0
7497.0
7538.0
100
%
0
mass
5000 6000 7000 8000 9000 10000 11000 12000 13000 14000 15000 16000 17000 18000 19000

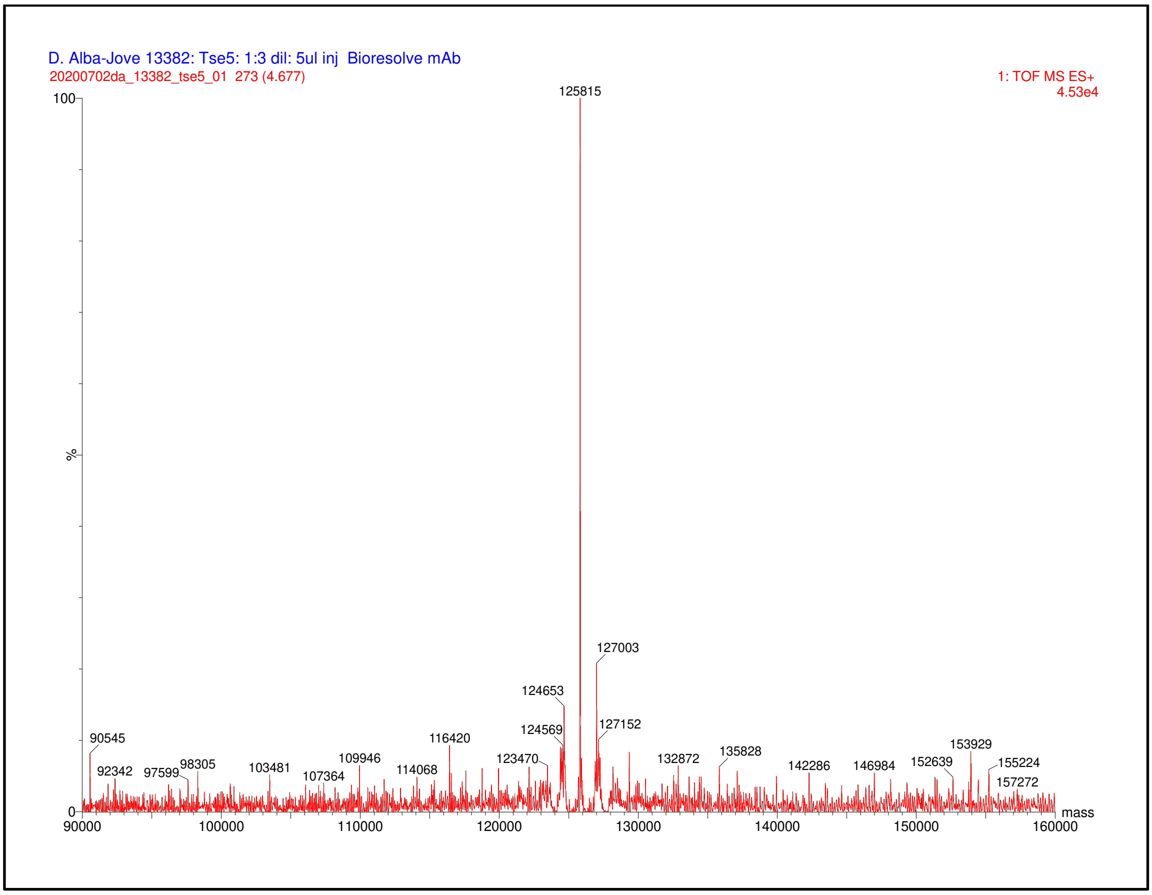

D. Alba-Jove 13382: Tse5: 1:3 dil: 5ul inj Bioresolve mAb
20200702da_13382_tse5_01 273 (4.677)
1: TOF MS ES+
4.53e4
125815
127003
124653
124569
127152
90545
92342
97599
98305
103481
107364
109946
114068
116420
123470
132872
135828
142286
146984
152639
153929
155224
157272
100
%
0
mass
90000
100000
110000
120000
130000
140000
150000
160000

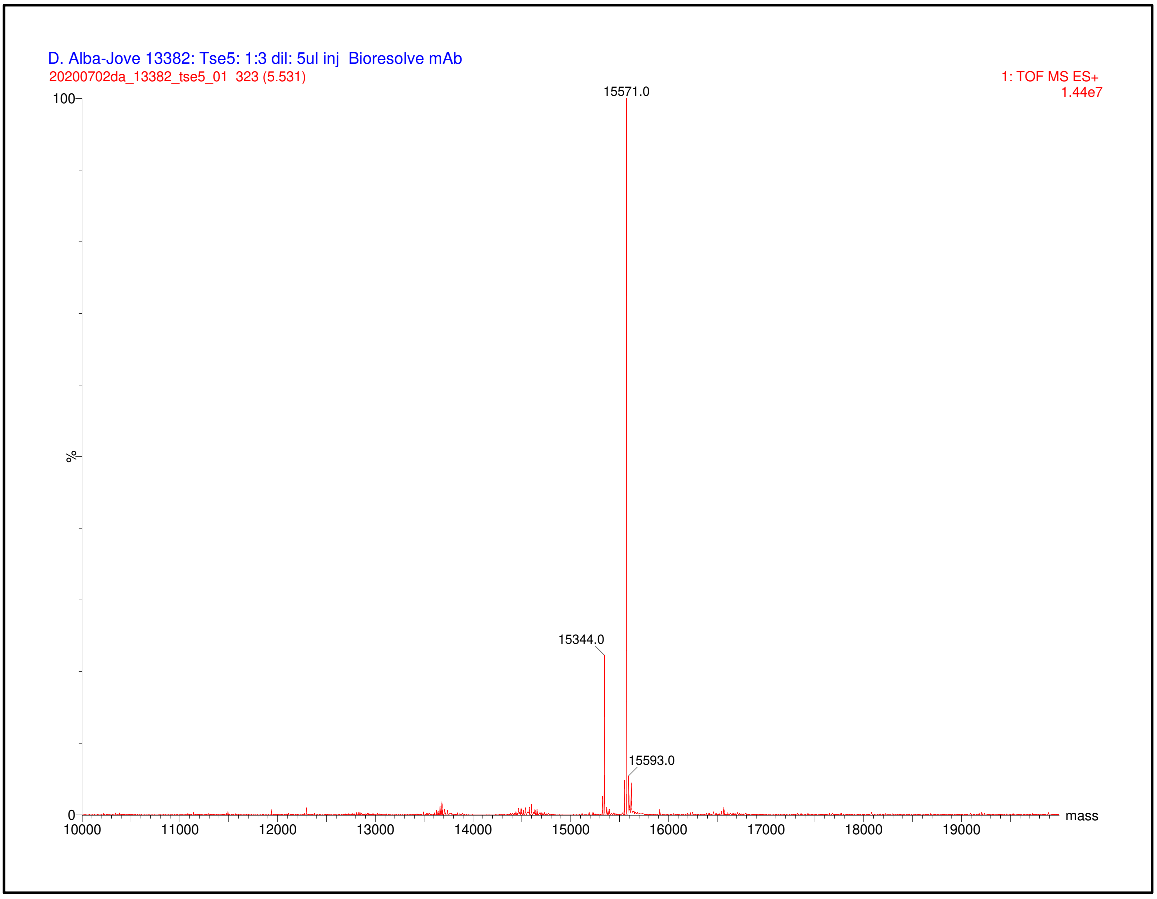

D. Alba-Jove 13382: Tse5: 1:3 dil: 5ul inj Bioresolve mAb
20200702da_13382_tse5_01 323 (5.531)
1: TOF MS ES+
1.44e7
15571.0
15344.0
15593.0
100
%
0
mass
10000
11000
12000
13000
14000
15000
16000
17000
18000
19000

Supplementary Note 3: N-terminal sequencing report Tse5-NT

SequencePro™

QUIMICA DE PROTEINAS (CIB-CSIC)
Ramiro de Maeztu, 9
28040 Madrid

PNA-SQP004-F02

SAMPLE INFORMATION

Sample Name:	David Albesa_Nterminal	Std Amount:	8.000 pmols
ID Code:		Sample Amount:	0.000 pmols
		Detector Scale:	0.005 AUFS
Comments:	MMGSS		

SEQUENCER INFORMATION

Name:	PROCISE	Model Number:	494
Method:	Pulsed liquid PVDF mod	Cartridge:	D
Operator:	Javier Varela		

Wednesday, March 09, 2022 09:58:48

David Albesa_Nterminal - 07Mar2022 13-04-34 - Page 1 of 8

Cycle 1:Blank 1

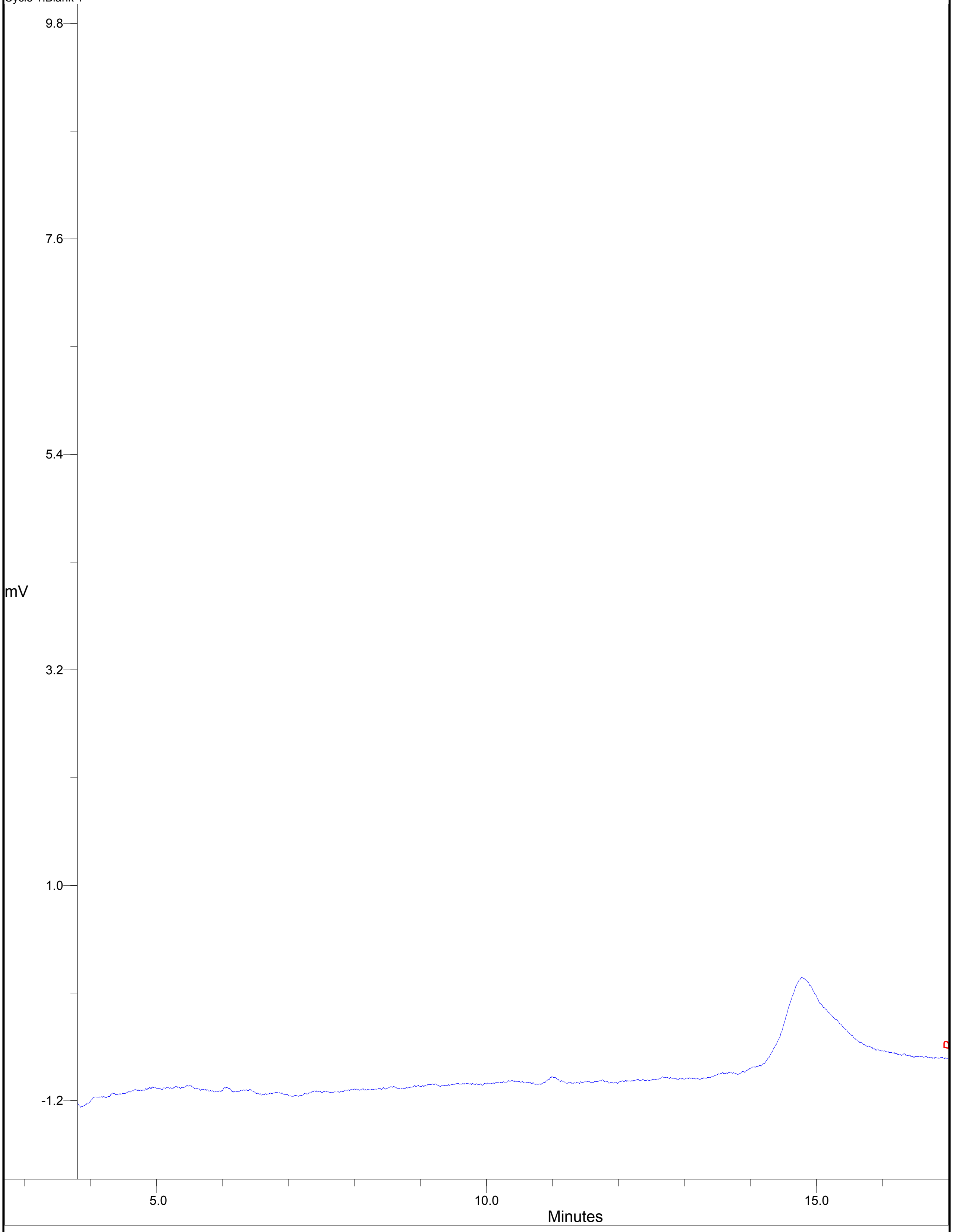

Cycle 2:Standard 1

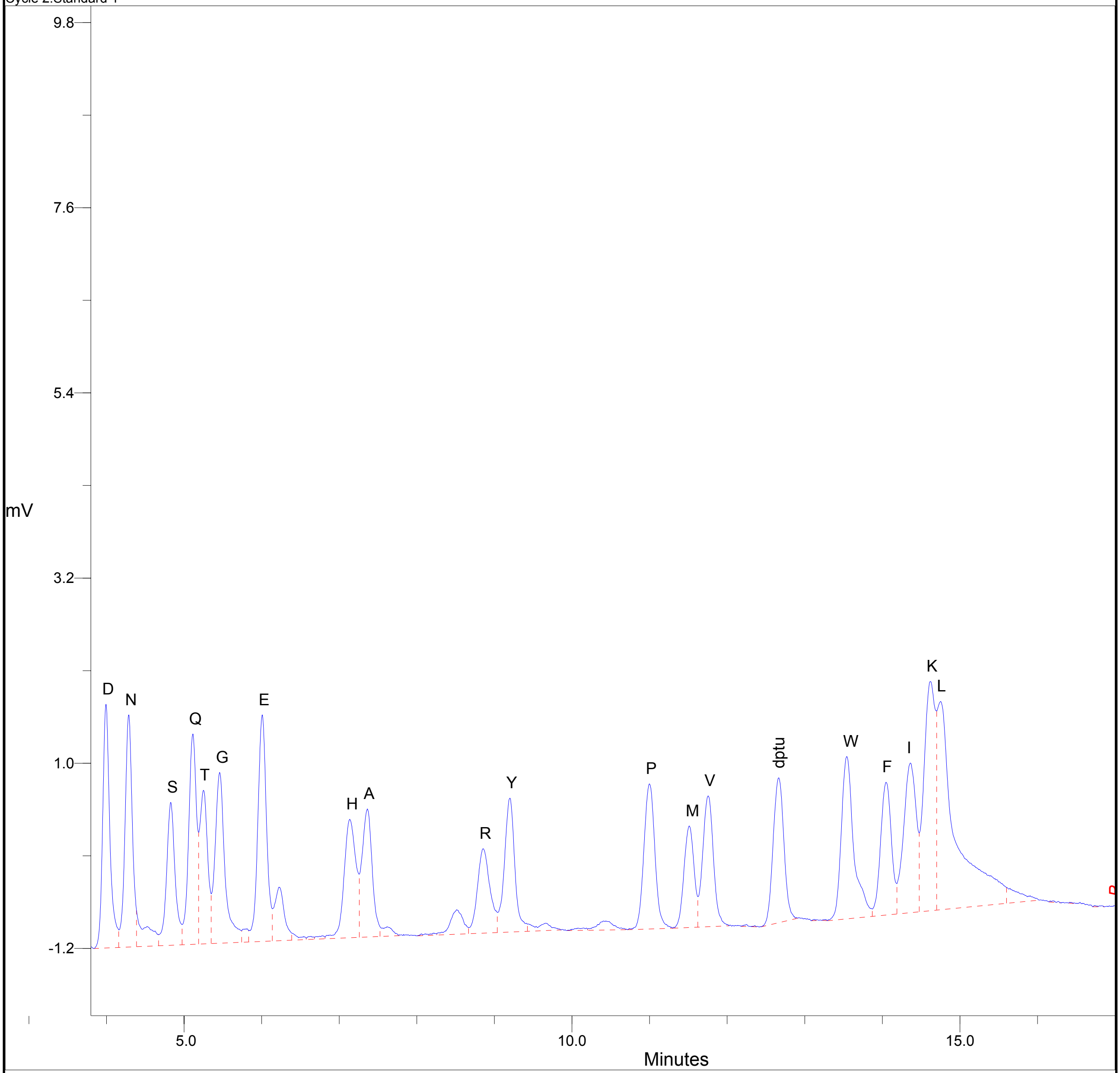

PEAK ID	R.TIME (mins)	C.TIME (mins)	HEIGHT (mV)	PMOL HT
D	3.99	3.99	2.900	8.000
N	4.29	4.29	2.760	8.000
	4.52		0.203	
S	4.83	4.83	1.698	8.000
Q	5.11	5.11	2.503	8.000
T	5.25	5.25	1.826	8.000
G	5.46	5.46	2.031	8.000
	5.81		0.159	
E	6.01	6.01	2.694	8.000
	6.22		0.638	
	6.54		0.033	
	6.64		0.038	
	6.79		0.038	
H	7.14	7.14	1.409	8.000
A	7.36	7.36	1.524	8.000
	7.62		0.087	
	8.05		0.023	
	8.22		0.026	
	8.51		0.292	
R	8.85	8.85	1.005	8.000
Y	9.20	9.20	1.592	8.000

PEAK ID	R.TIME (mins)	C.TIME (mins)	HEIGHT (mV)	PMOL HT
	9.65		0.085	
	10.15		0.028	
	10.44		0.108	
P	11.00	11.00	1.726	8.000
M	11.51	11.51	1.209	8.000
V	11.75	11.75	1.556	8.000
	12.25		0.025	
dptu	12.66	12.66	1.722	8.000
	13.12		0.018	
	13.27		0.015	
W	13.54	13.54	1.930	8.000
F	14.05	14.05	1.579	8.000
I	14.36	14.36	1.778	8.000
K	14.62	14.62	2.725	8.000
L	14.75	14.75	2.476	8.000
	15.90		0.033	
	16.20		0.018	
	16.27		0.015	
	16.42		0.011	
	16.74		0.016	
	16.84		0.014	

SequencePro™

QUIMICA DE PROTEINAS (CIB-CSIC)
Ramiro de Maeztu, 9
28040 Madrid

PNA-SQP004-F02

Cycle 3:

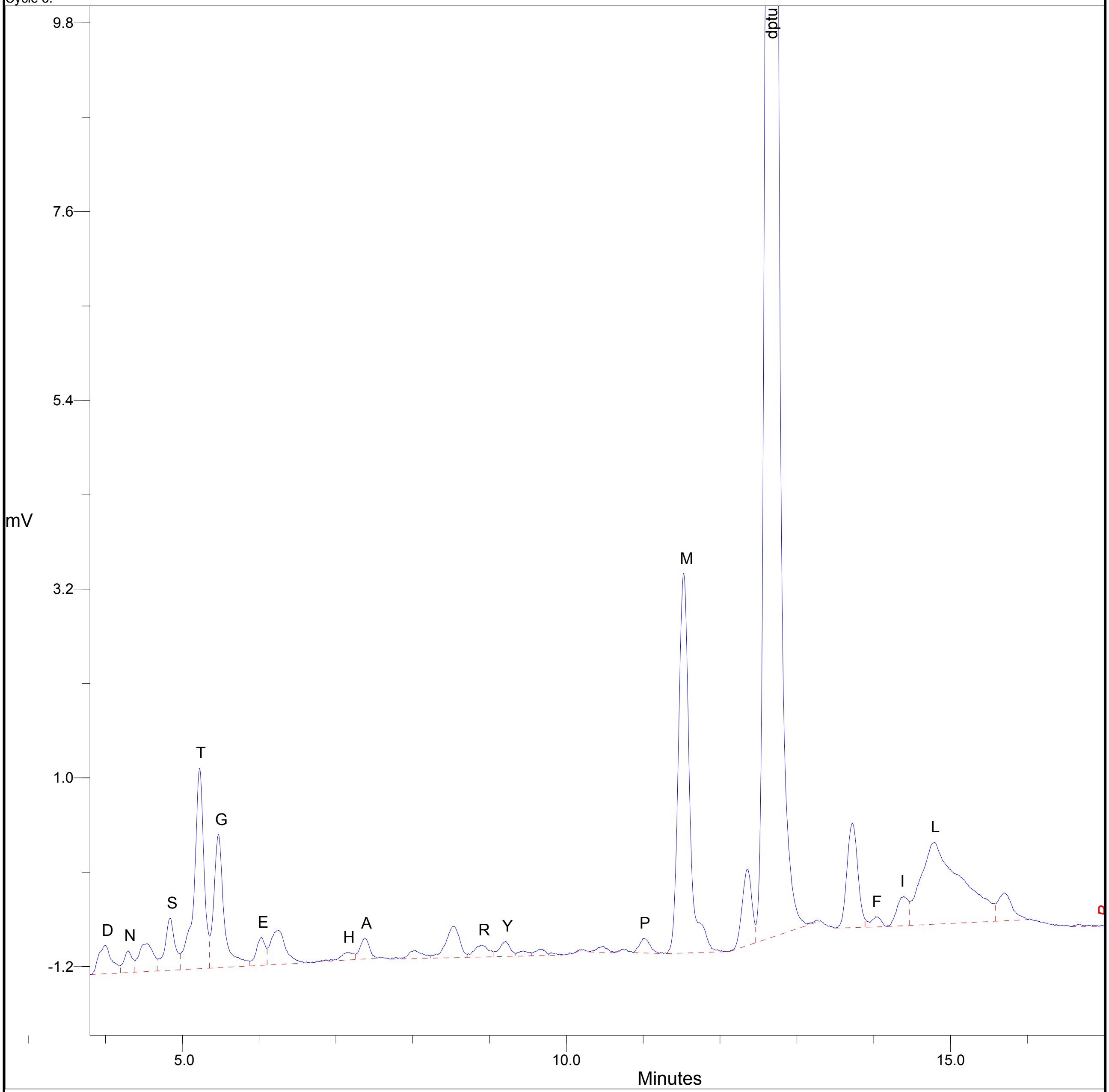

PEAK ID	R.TIME (mins)	C.TIME (mins)	HEIGHT (mV)	PMOL HT
D	4.00	3.99	0.332	0.916
N	4.29	4.29	0.255	0.738
	4.54		0.324	
S	4.85	4.83	0.607	2.860
T	5.22	5.25	2.341	10.256
G	5.47	5.46	1.555	6.126
E	6.03	6.01	0.328	0.975
	6.24		0.402	
	6.75		0.012	
	6.83		0.013	
	6.93		0.023	
H	7.14	7.14	0.089	0.505
A	7.38	7.36	0.243	1.277
	7.78		0.022	
	8.04		0.095	
	8.54		0.366	
R	8.90	8.85	0.139	1.109
Y	9.21	9.20	0.178	0.893
	9.44		0.062	

PEAK ID	R.TIME (mins)	C.TIME (mins)	HEIGHT (mV)	PMOL HT
	9.68		0.076	
	9.80		0.033	
	10.04		0.015	
	10.18		0.011	
	10.48		0.072	
	10.72		0.020	
P	11.00	11.00	0.170	0.791
M	11.53	11.51	4.426	29.292
	12.35		0.889	
dptu	12.67	12.66	29.603	137.511
	13.26		0.022	
	13.73		1.214	
F	14.03	14.05	0.112	0.570
I	14.39	14.36	0.343	1.544
L	14.79	14.75	0.957	3.093
	15.71		0.327	
	16.65		0.030	
	16.84		0.020	

Wednesday, March 09, 2022 09:58:50

David Albesa_Nterminal - 07Mar2022 13-04-34 - Page 4 of 8

Cycle 4:

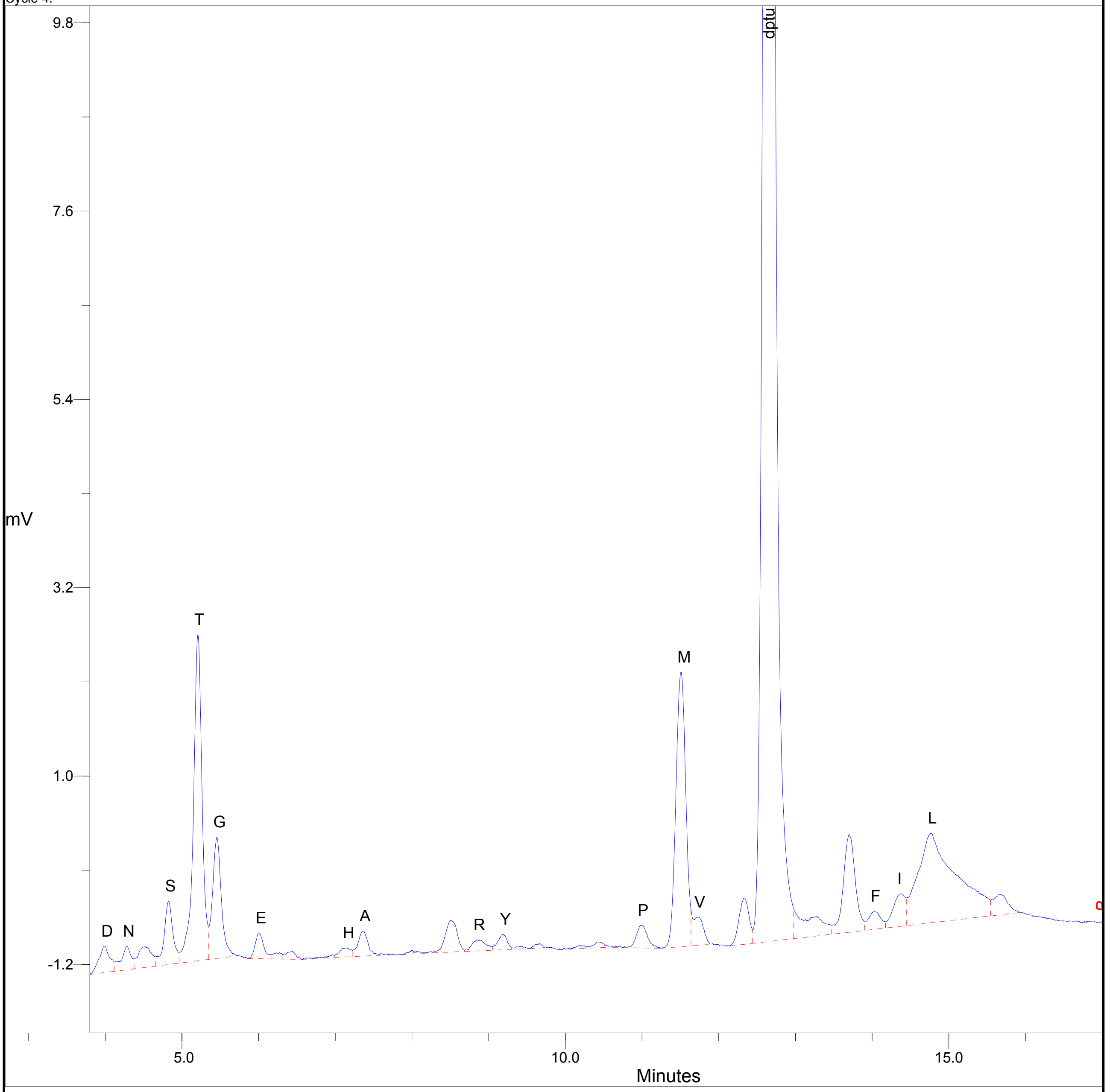

PEAK ID	R.TIME (mins)	C.TIME (mins)	HEIGHT (mV)	PMOL HT
D	4.00	3.99	0.309	0.854
N	4.28	4.29	0.276	0.801
	4.52		0.244	
S	4.83	4.83	0.740	3.487
T	5.21	5.25	3.813	16.705
G	5.45	5.46	1.419	5.589
E	6.01	6.01	0.302	0.898
	6.27		0.075	
	6.45		0.094	
	6.63		0.017	
	6.96		0.037	
H	7.15	7.14	0.108	0.616
A	7.37	7.36	0.295	1.550
	7.60		0.026	
	7.67		0.016	
	8.00		0.035	
	8.24		0.016	
	8.52		0.375	
R	8.85	8.85	0.130	1.037

PEAK ID	R.TIME (mins)	C.TIME (mins)	HEIGHT (mV)	PMOL HT
Y	9.20	9.20	0.182	0.913
	9.42		0.032	
	9.66		0.054	
	9.98		0.015	
	10.19		0.036	
	10.43		0.069	
	10.65		0.023	
P	10.99	11.00	0.267	1.237
M	11.51	11.51	3.207	21.229
V	11.73	11.75	0.233	1.200
	12.33		0.537	
dptu	12.65	12.66	27.315	126.885
	13.28		0.184	
	13.70		1.086	
F	14.03	14.05	0.144	0.731
I	14.38	14.36	0.317	1.424
L	14.78	14.75	1.001	3.235
	15.67		0.230	
	16.78		0.020	

Cycle 5:

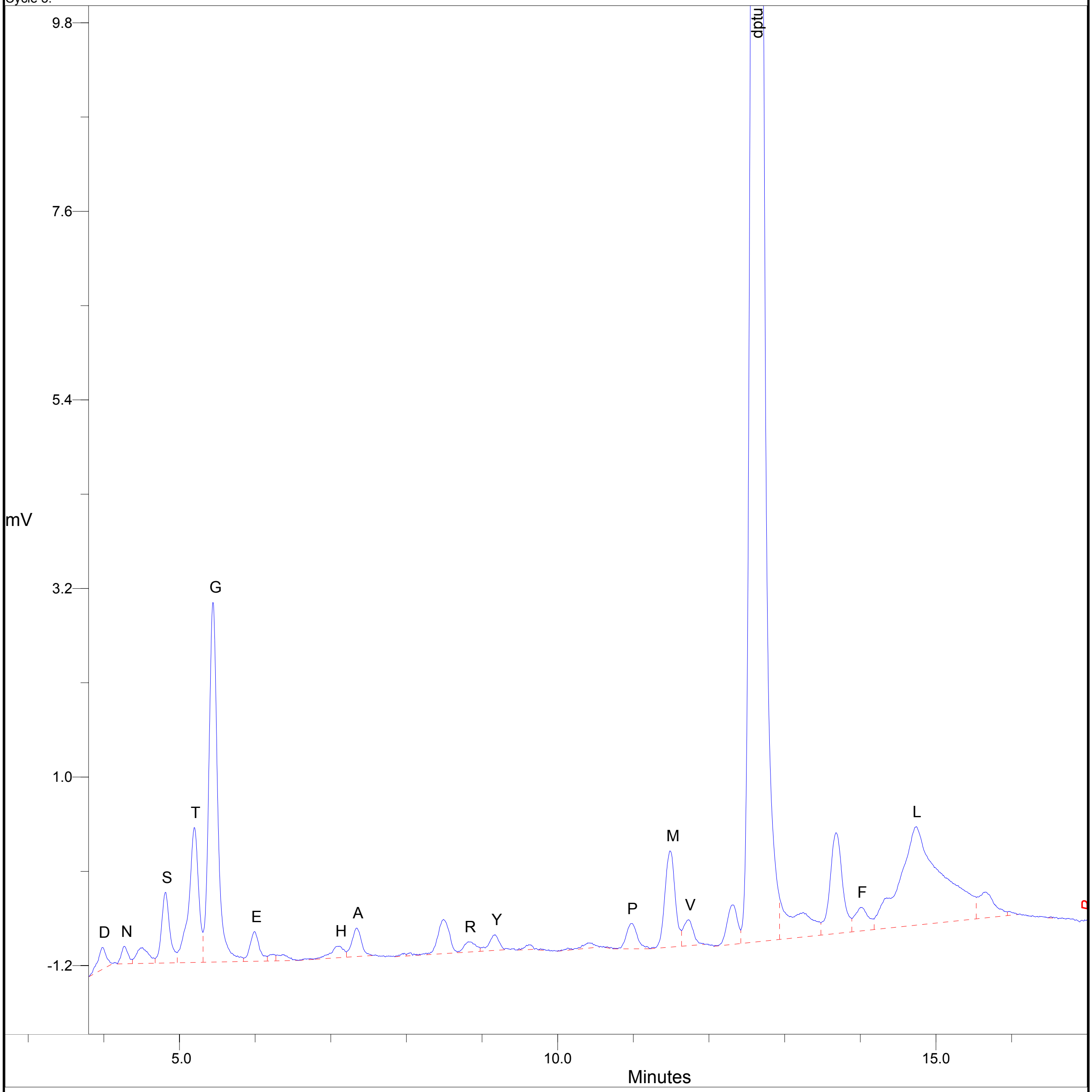

PEAK ID	R.TIME (mins)	C.TIME (mins)	HEIGHT (mV)	PMOL HT
D	3.98	3.99	0.256	0.705
N	4.28	4.29	0.204	0.592
	4.51		0.184	
S	4.81	4.83	0.826	3.889
T	5.20	5.25	1.574	6.897
G	5.44	5.46	4.198	16.536
E	6.00	6.01	0.347	1.031
	6.24		0.078	
	6.38		0.072	
	6.72		0.013	
H	7.11	7.14	0.134	0.761
A	7.34	7.36	0.333	1.748
	7.97		0.035	
	8.05		0.035	
	8.49		0.400	
R	8.82	8.85	0.121	0.962
Y	9.17	9.20	0.184	0.923
	9.32		0.015	

PEAK ID	R.TIME (mins)	C.TIME (mins)	HEIGHT (mV)	PMOL HT
	9.62		0.058	
	9.77		0.016	
	10.13		0.026	
	10.40		0.064	
	10.65		0.012	
P	10.97	11.00	0.296	1.371
M	11.48	11.51	1.124	7.442
V	11.73	11.75	0.304	1.563
	12.31		0.462	
dptu	12.63	12.66	29.656	137.759
	13.25		0.279	
	13.68		1.177	
F	14.01	14.05	0.276	1.398
L	14.74	14.75	1.150	3.716
	15.65		0.300	
	15.97		0.049	
	16.52		0.016	

Cycle 6:

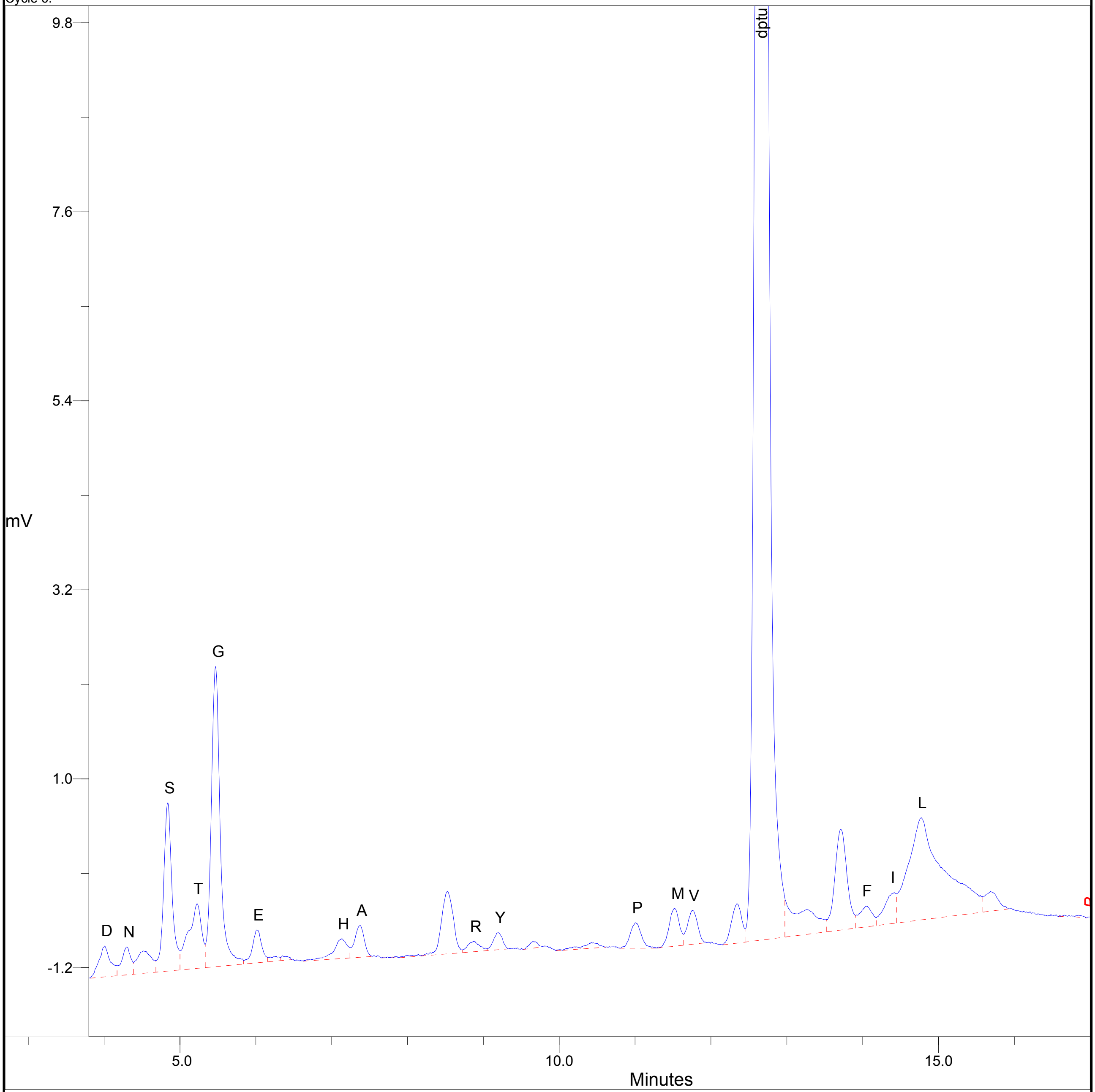

PEAK ID	R.TIME (mins)	C.TIME (mins)	HEIGHT (mV)	PMOL HT
D	4.01	3.99	0.362	0.998
N	4.30	4.29	0.325	0.943
	4.51		0.262	
S	4.84	4.83	1.960	9.231
T	5.22	5.25	0.753	3.298
G	5.47	5.46	3.495	13.764
E	6.01	6.01	0.383	1.138
	6.25		0.057	
	6.35		0.054	
H	7.13	7.14	0.230	1.309
A	7.38	7.36	0.371	1.949
	7.70		0.011	
	7.86		0.017	
	7.91		0.014	
	8.09		0.019	
	8.53		0.727	
R	8.87	8.85	0.122	0.971
Y	9.20	9.20	0.199	1.001

PEAK ID	R.TIME (mins)	C.TIME (mins)	HEIGHT (mV)	PMOL HT
	9.65		0.078	
	10.01		0.013	
	10.22		0.032	
	10.43		0.066	
P	11.01	11.00	0.297	1.375
M	11.53	11.51	0.440	2.916
V	11.75	11.75	0.393	2.023
	12.34		0.460	
dptu	12.67	12.66	31.142	144.662
	13.27		0.280	
	13.71		1.177	
F	14.04	14.05	0.244	1.234
I	14.42	14.36	0.358	1.612
L	14.78	14.75	1.190	3.846
	15.69		0.229	
	16.66		0.017	
	16.85		0.025	

Cycle 7:

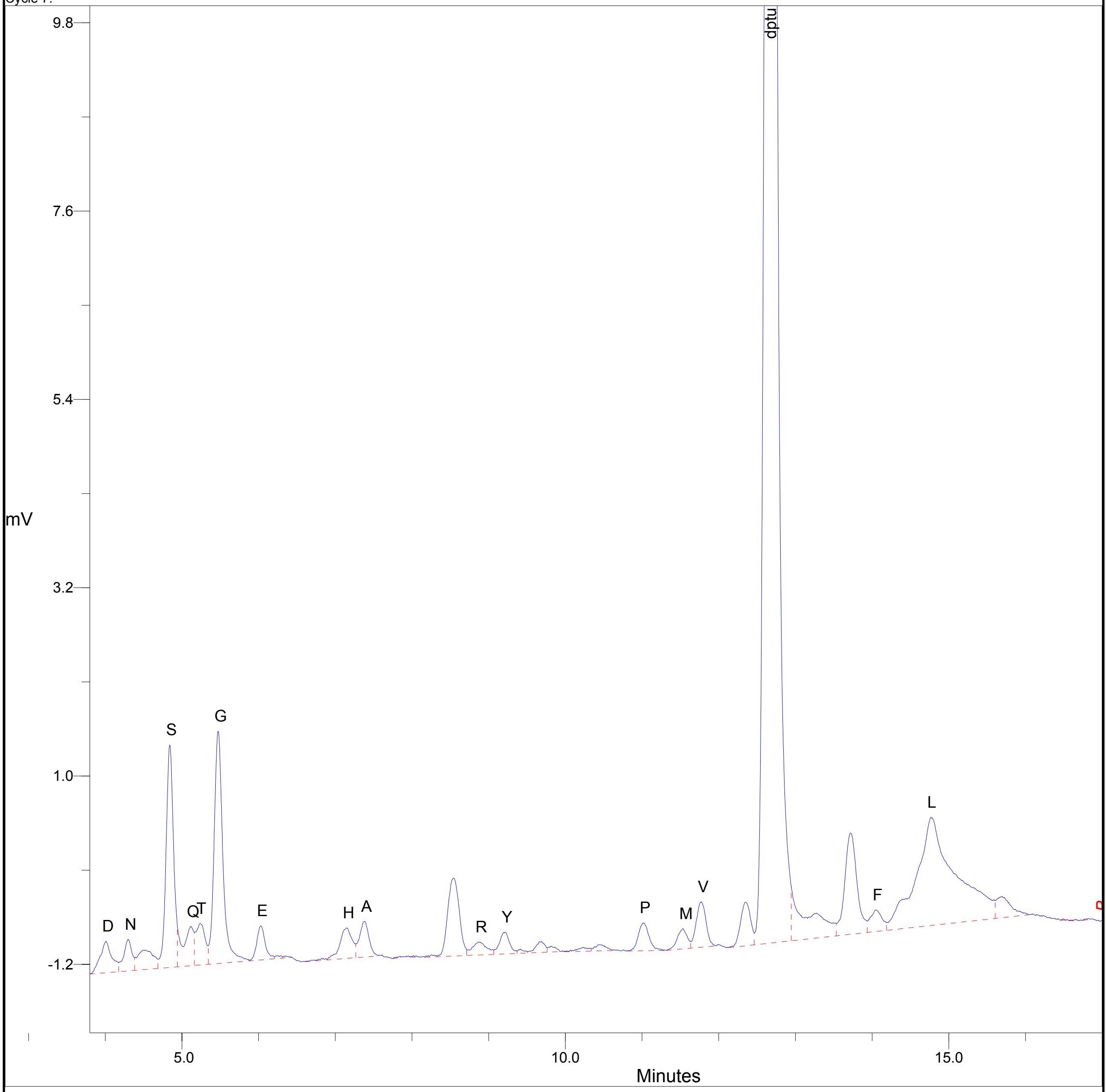

PEAK ID	R.TIME (mins)	C.TIME (mins)	HEIGHT (mV)	PMOL HT
D	4.01	3.99	0.368	1.016
N	4.30	4.29	0.371	1.074
	4.52		0.230	
S	4.84	4.83	2.599	12.245
Q	5.11	5.11	0.446	1.425
T	5.24	5.25	0.486	2.127
G	5.47	5.46	2.715	10.692
E	6.03	6.01	0.398	1.182
	6.29		0.031	
	6.36		0.020	
	6.82		0.024	
H	7.15	7.14	0.357	2.027
A	7.38	7.36	0.416	2.184
	7.84		0.013	
	8.02		0.018	
	8.26		0.024	
	8.54		0.913	
R	8.88	8.85	0.146	1.166
Y	9.22	9.20	0.254	1.278

PEAK ID	R.TIME (mins)	C.TIME (mins)	HEIGHT (mV)	PMOL HT
	9.41		0.051	
	9.69		0.127	
	9.82		0.069	
	10.07		0.021	
	10.23		0.047	
	10.44		0.073	
P	11.02	11.00	0.327	1.514
M	11.53	11.51	0.238	1.573
V	11.78	11.75	0.532	2.735
	12.35		0.510	
dptu	12.68	12.66	31.074	144.346
	13.28		0.285	
	13.72		1.183	
F	14.05	14.05	0.252	1.278
L	14.77	14.75	1.265	4.087
	15.69		0.250	
	16.50		0.016	
	16.66		0.020	
	16.82		0.012	

Supplementary Note 4: N-terminal sequencing report Tse5-Shell

SequencePro™

QUIMICA DE PROTEINAS (CIB-CSIC)
Ramiro de Maeztu, 9
28040 Madrid

PNA-SQP004-F02

SAMPLE INFORMATION

Sample Name:	David Albesa_Shell	Std Amount:	8.000 pmols
ID Code:		Sample Amount:	0.000 pmols
		Detector Scale:	0.005 AUFS
Comments:	PVNPM		

SEQUENCER INFORMATION

Name:	PROCISE	Model Number:	494
Method:	Pulsed liquid PVDF mod	Cartridge:	D
Operator:	Javier Varela		

Wednesday, January 18, 2023 16:36:15 David Albesa_Shell - 18Jan2023 09-51-06 - Page 1 of 8

Cycle 1:Blank 1

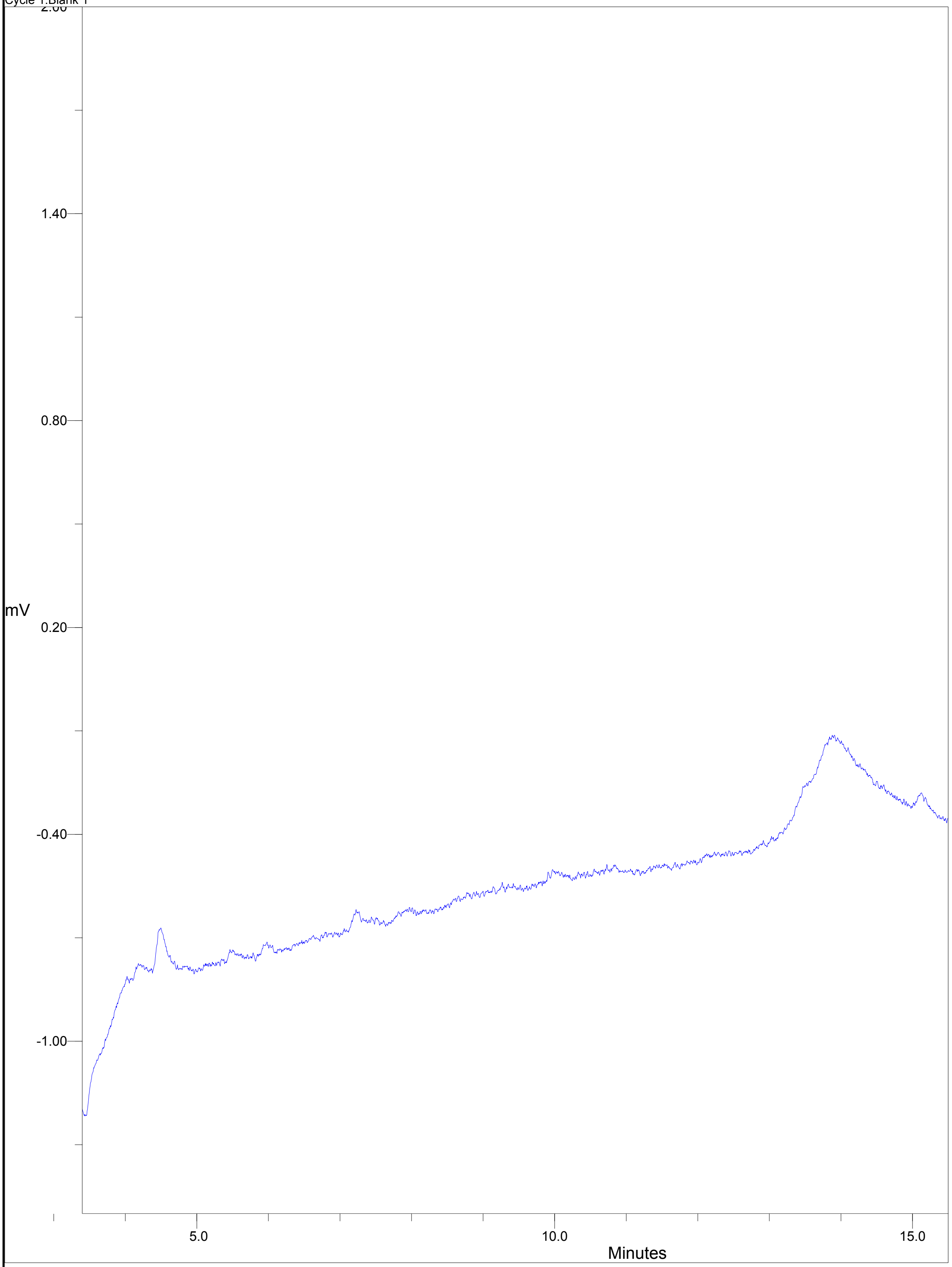

Cycle 2:

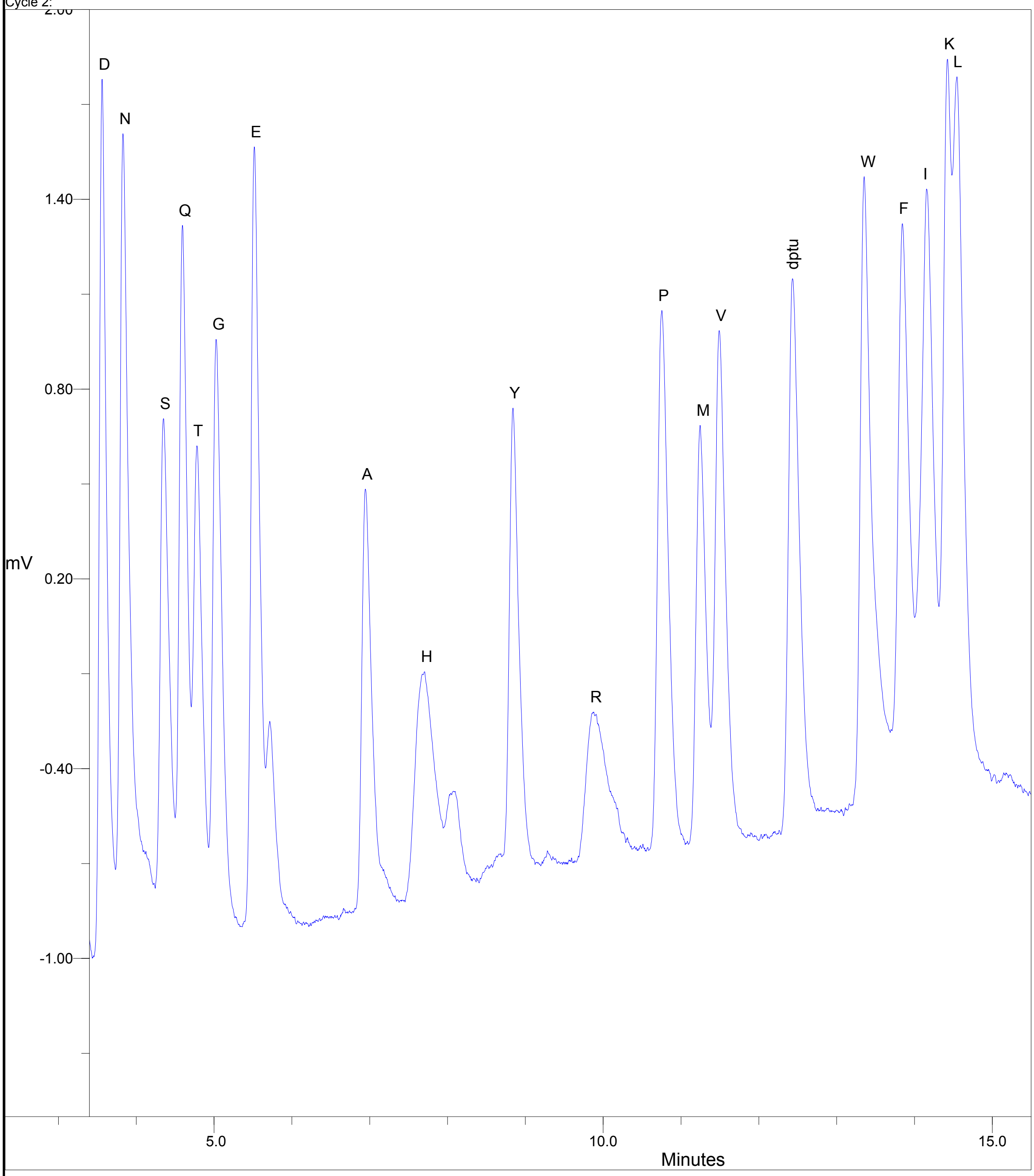

PEAK ID	R.TIME (mins)	C.TIME (mins)	HEIGHT (mV)	PMOL HT	PEAK ID	R.TIME (mins)	C.TIME (mins)	HEIGHT (mV)	PMOL HT
D	3.56	3.56	2.776	8.000	R	9.88	9.88	0.453	8.000
N	3.83	3.83	2.591	8.000	P	10.75	10.75	1.706	8.000
S	4.35	4.35	1.669	8.000	M	11.25	11.25	1.330	8.000
Q	4.59	4.59	2.271	8.000	V	11.49	11.49	1.624	8.000
T	4.78	4.78	1.565	8.000	dptu	12.43	12.43	1.734	8.000
G	5.03	5.03	1.892	8.000	W	13.35	13.35	1.996	8.000
E	5.52	5.52	2.480	8.000	F	13.85	13.85	1.802	8.000
A	6.95	6.95	1.338	8.000	I	14.16	14.16	1.882	8.000
H	7.70	7.70	0.706	8.000	K	14.43	14.43	2.268	8.000
Y	8.84	8.84	1.466	8.000	L	14.55	14.55	2.200	8.000

SequencePro™

QUIMICA DE PROTEINAS (CIB-CSIC)
Ramiro de Maeztu, 9
28040 Madrid

PNA-SQP004-F02

Cycle 3:

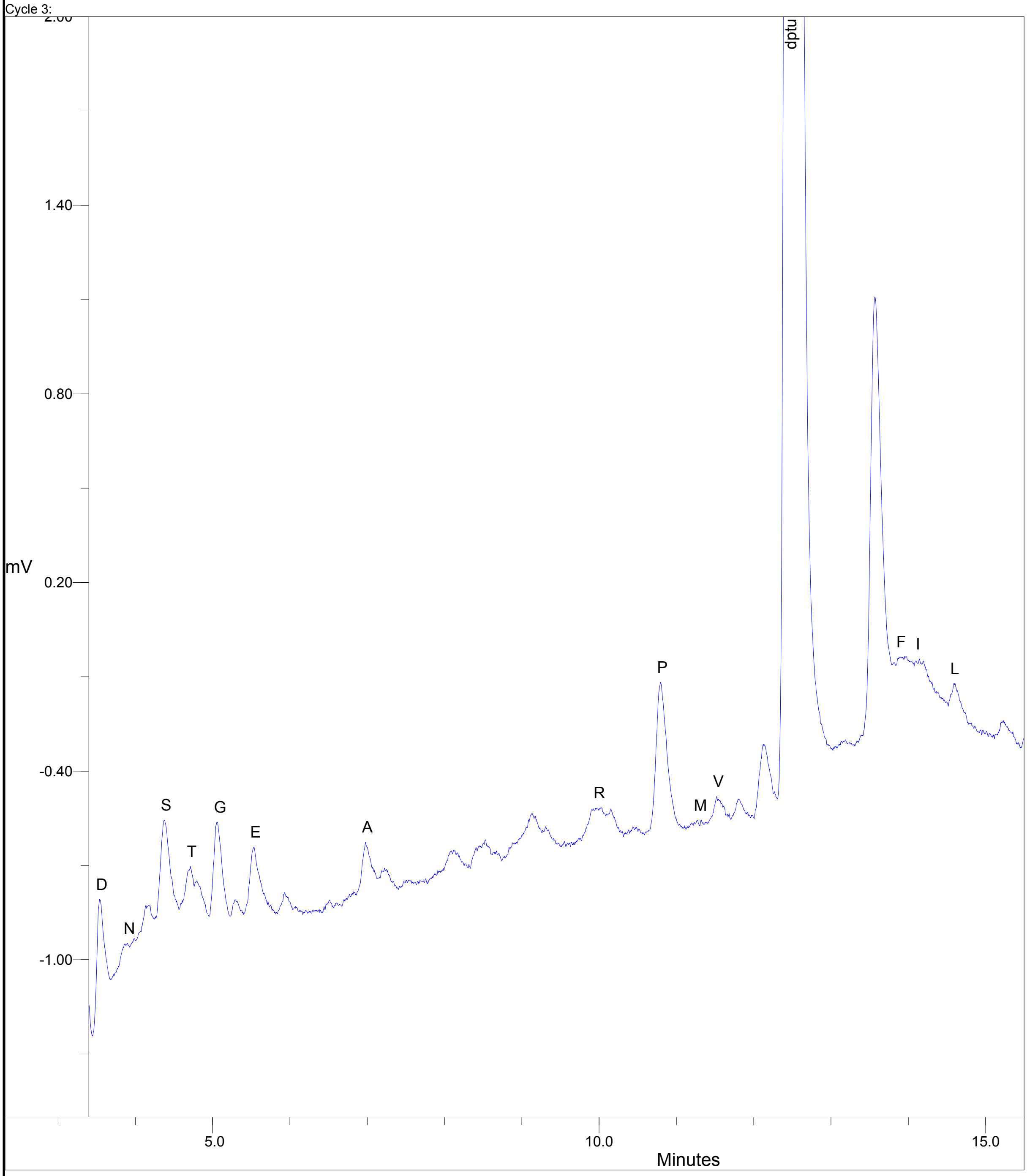

PEAK ID	R.TIME (mins)	C.TIME (mins)	HEIGHT (mV)	PMOL HT
D	3.54	3.56	0.402	1.159
N	3.90	3.83	0.134	0.413
S	4.38	4.35	0.354	1.697
T	4.71	4.78	0.083	0.423
G	5.06	5.03	0.299	1.265
E	5.53	5.52	0.219	0.705
A	6.98	6.95	0.182	1.091
R	9.97	9.88	0.017	0.307
P	10.80	10.75	0.477	2.239
M	11.27	11.25	0.021	0.128

PEAK ID	R.TIME (mins)	C.TIME (mins)	HEIGHT (mV)	PMOL HT
V	11.52	11.49	0.083	0.410
dptu	12.48	12.43	20.587	94.992
F	13.89	13.85	0.028	0.126
I	14.14	14.16	0.019	0.082
L	14.59	14.55	0.089	0.323

SequencePro™

QUIMICA DE PROTEINAS (CIB-CSIC)
Ramiro de Maeztu, 9
28040 Madrid

PNA-SQP004-F02

Cycle 4:

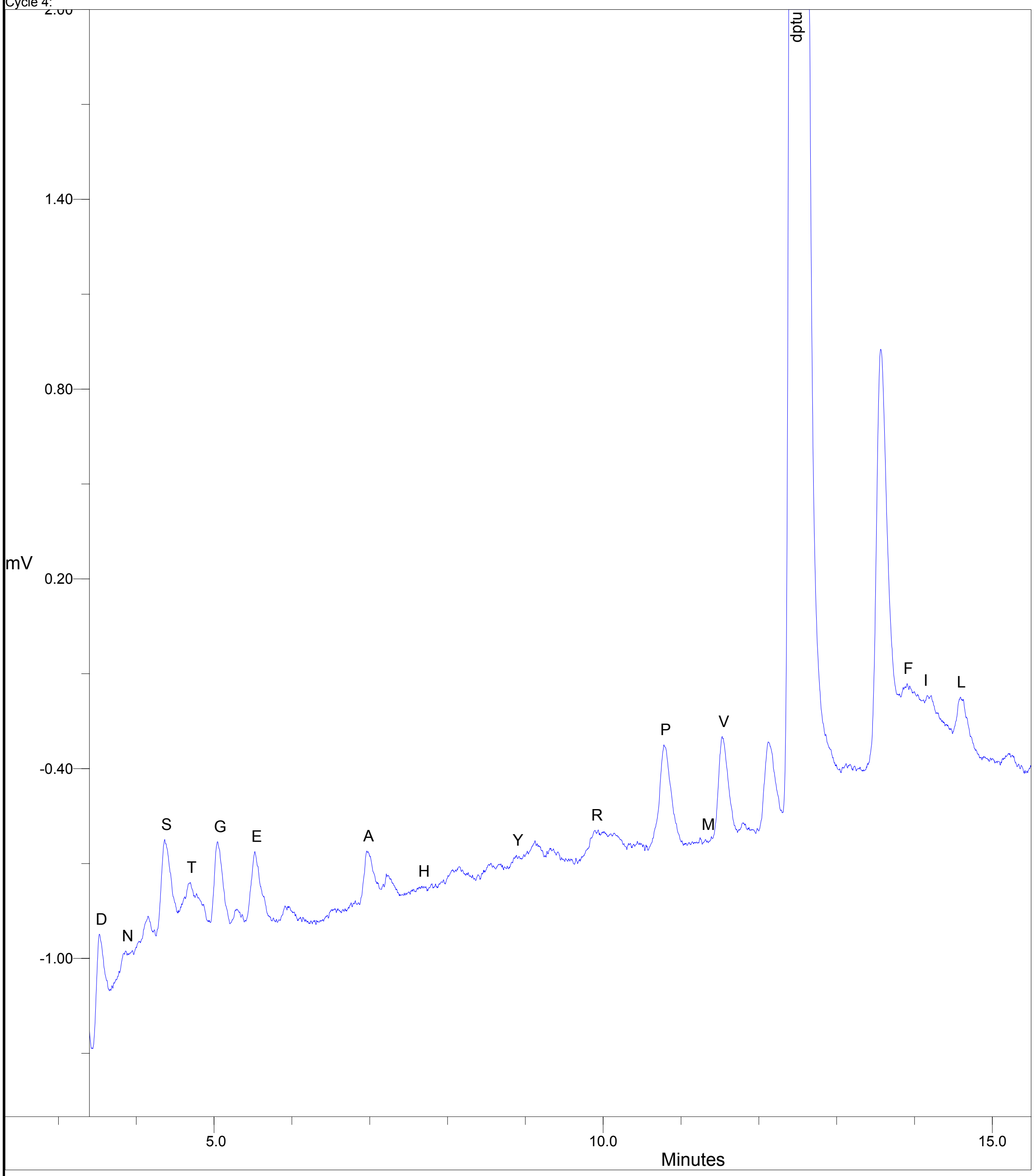

PEAK ID	R.TIME (mins)	C.TIME (mins)	HEIGHT (mV)	PMOL HT
D	3.52	3.56	0.332	0.958
N	3.86	3.83	0.157	0.483
S	4.37	4.35	0.327	1.566
T	4.70	4.78	0.070	0.360
G	5.05	5.03	0.257	1.088
E	5.53	5.52	0.224	0.722
A	6.96	6.95	0.185	1.107
H	7.67	7.70	0.021	0.241
Y	8.88	8.84	0.053	0.289
R	9.89	9.88	0.019	0.339

PEAK ID	R.TIME (mins)	C.TIME (mins)	HEIGHT (mV)	PMOL HT
P	10.78	10.75	0.327	1.535
M	11.31	11.25	0.014	0.084
V	11.53	11.49	0.328	1.616
dptu	12.48	12.43	20.392	94.089
F	13.90	13.85	0.070	0.310
I	14.16	14.16	0.034	0.145
L	14.59	14.55	0.139	0.505

Wednesday, January 18, 2023 16:36:17

David Albesa_Shell - 18Jan2023 09-51-06 - Page 5 of 8

SequencePro™

QUIMICA DE PROTEINAS (CIB-CSIC)
Ramiro de Maeztu, 9
28040 Madrid

PNA-SQP004-F02

Cycle 5:

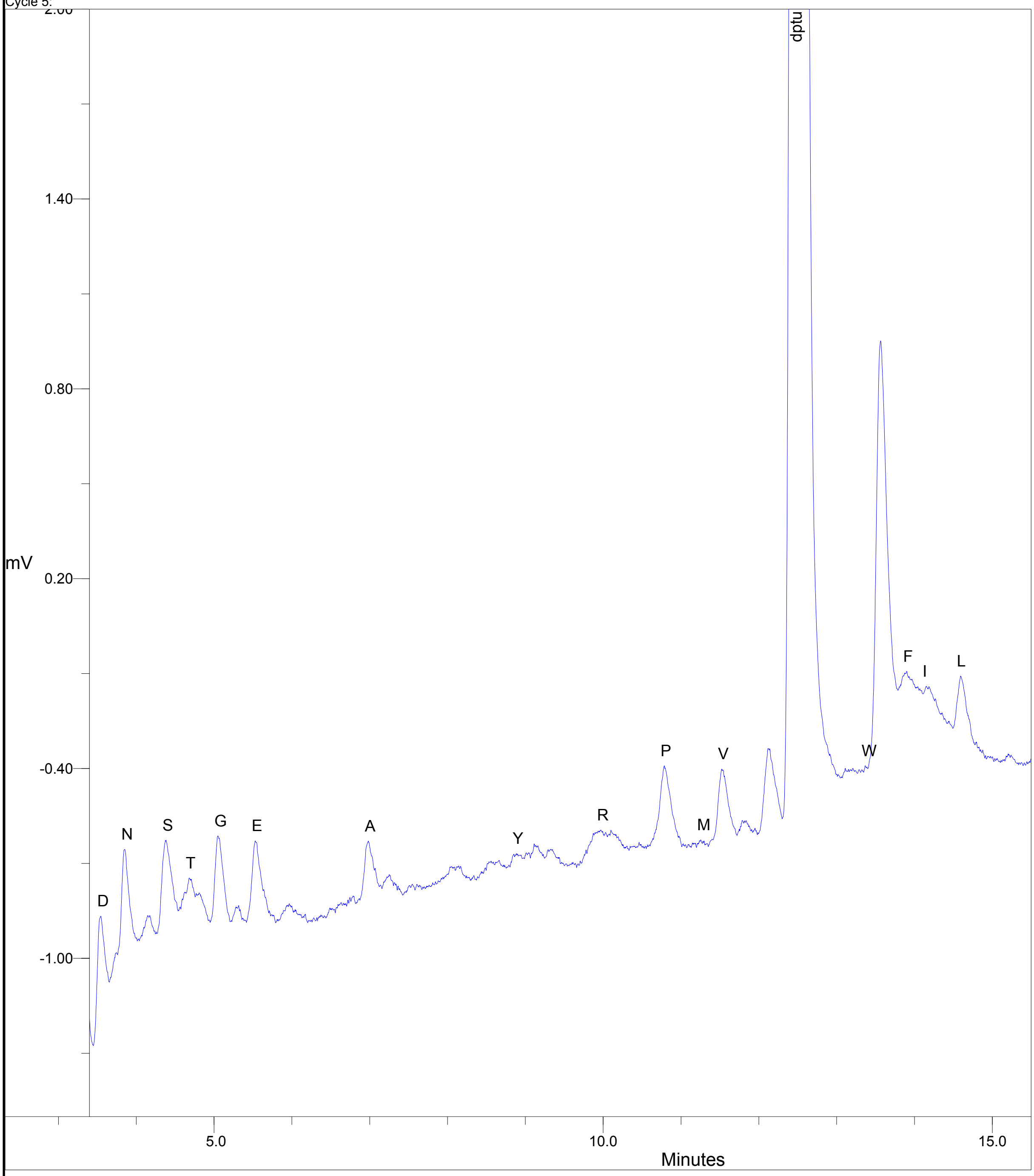

PEAK ID	R.TIME (mins)	C.TIME (mins)	HEIGHT (mV)	PMOL HT
D	3.55	3.56	0.376	1.083
N	3.85	3.83	0.476	1.471
S	4.38	4.35	0.314	1.504
T	4.68	4.78	0.087	0.444
G	5.05	5.03	0.273	1.157
E	5.53	5.52	0.259	0.835
A	6.98	6.95	0.170	1.018
Y	8.88	8.84	0.045	0.248
R	9.97	9.88	0.089	1.567
P	10.78	10.75	0.260	1.219

PEAK ID	R.TIME (mins)	C.TIME (mins)	HEIGHT (mV)	PMOL HT
M	11.25	11.25	0.024	0.143
V	11.52	11.49	0.229	1.130
dptu	12.48	12.43	20.417	94.206
W	13.37	13.35	0.015	0.062
F	13.90	13.85	0.086	0.381
I	14.15	14.16	0.035	0.148
L	14.59	14.55	0.185	0.671

Wednesday, January 18, 2023 16:36:17

David Albesa_Shell - 18Jan2023 09-51-06 - Page 6 of 8

QUIMICA DE PROTEINAS (CIB-CSIC)
Ramiro de Maeztu, 9
28040 Madrid

PNA-SQP004-F02

Cycle 6:

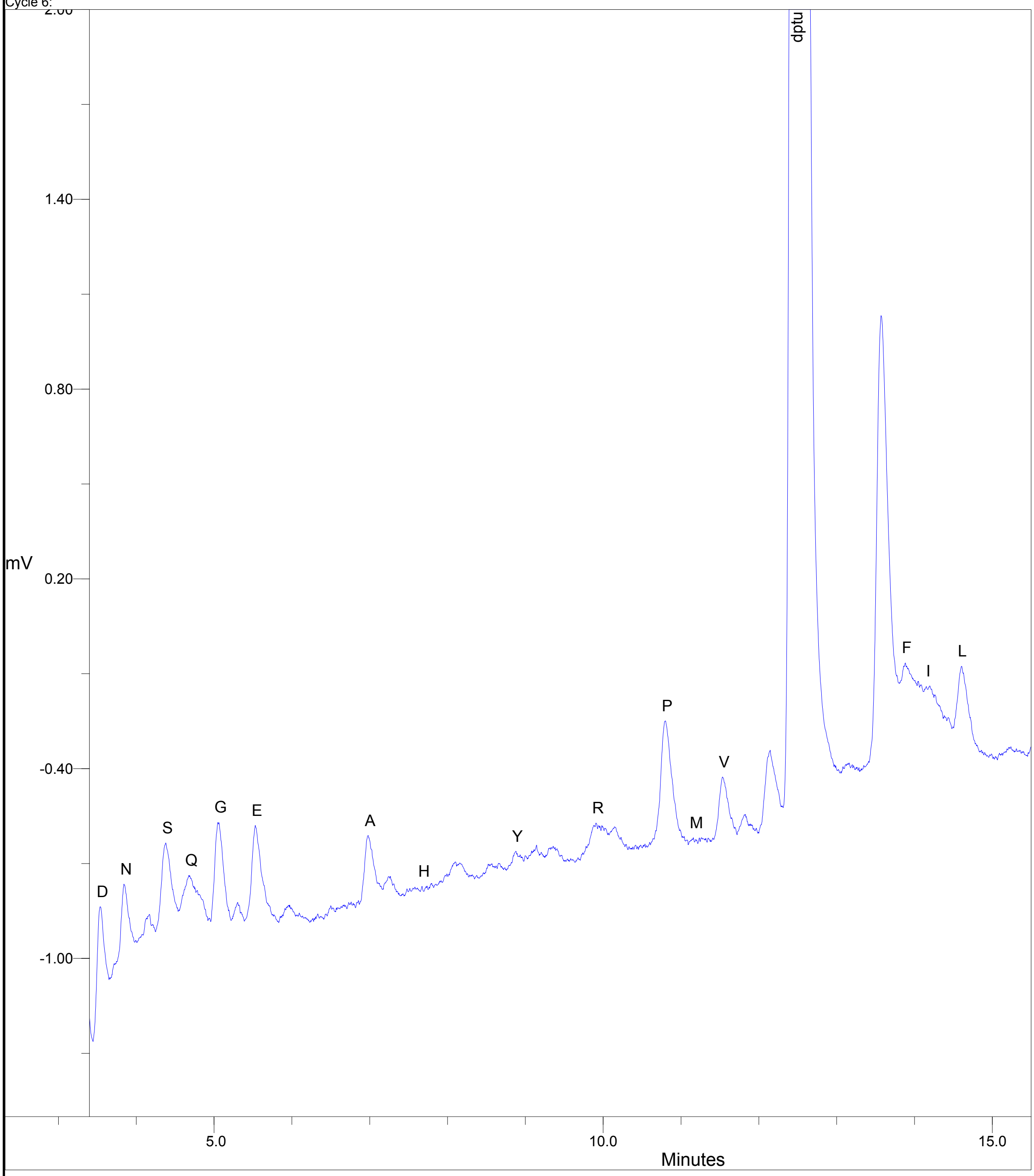

PEAK ID	R.TIME (mins)	C.TIME (mins)	HEIGHT (mV)	PMOL HT
D	3.54	3.56	0.413	1.190
N	3.84	3.83	0.439	1.354
S	4.38	4.35	0.487	2.334
Q	4.67	4.59	0.339	1.194
G	5.05	5.03	0.450	1.901
E	5.53	5.52	0.367	1.185
A	6.98	6.95	0.223	1.332
H	7.67	7.70	0.018	0.203
Y	8.88	8.84	0.036	0.195
R	9.90	9.88	0.048	0.850

PEAK ID	R.TIME (mins)	C.TIME (mins)	HEIGHT (mV)	PMOL HT
P	10.80	10.75	0.392	1.837
M	11.16	11.25	0.021	0.124
V	11.53	11.49	0.198	0.977
dptu	12.49	12.43	21.189	97.768
F	13.88	13.85	0.186	0.825
I	14.20	14.16	0.131	0.556
L	14.60	14.55	0.223	0.812

QUIMICA DE PROTEINAS (CIB-CSIC)
Ramiro de Maeztu, 9
28040 Madrid

PNA-SQP004-F02

Cycle 7:

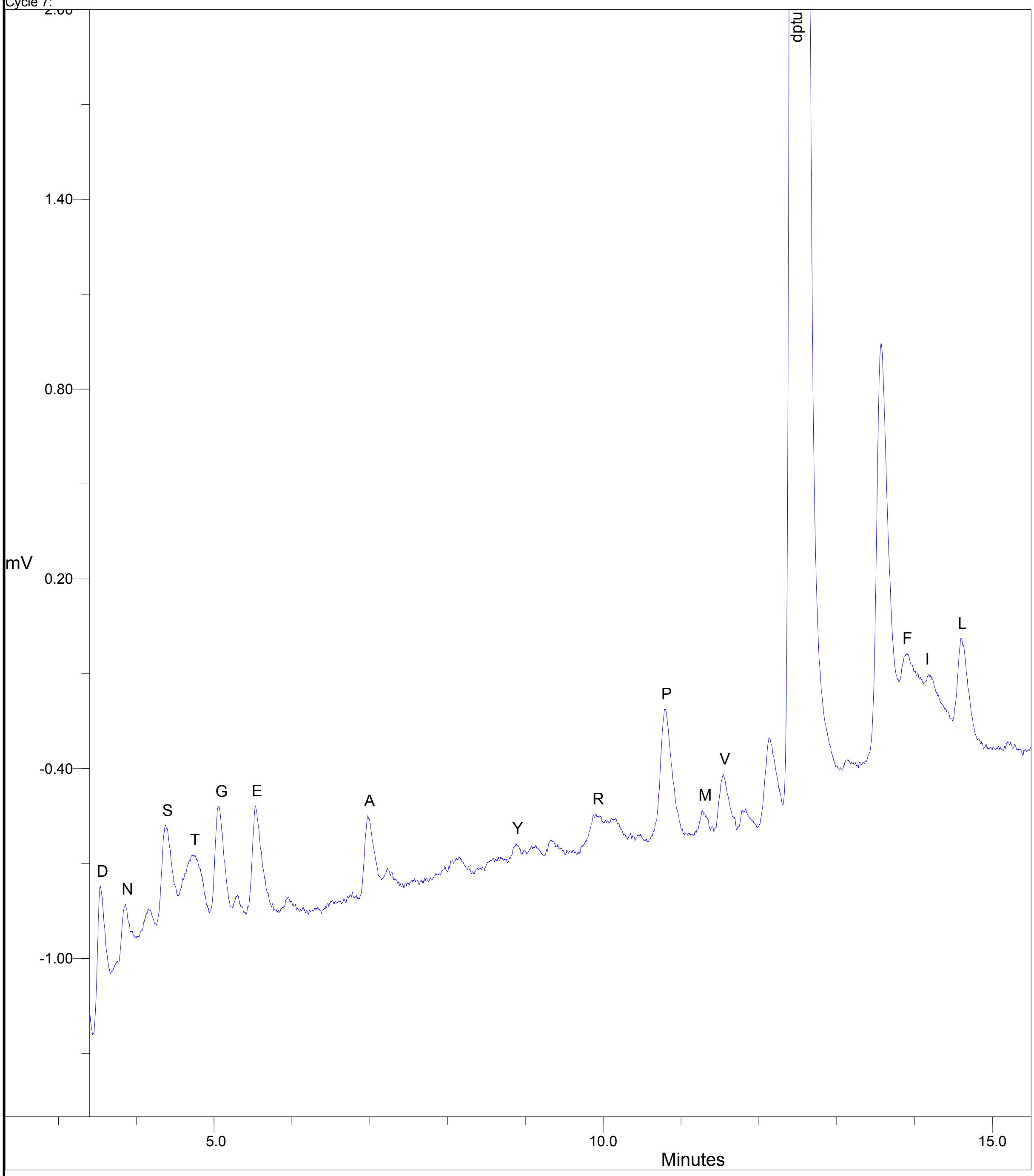

PEAK ID	R.TIME (mins)	C.TIME (mins)	HEIGHT (mV)	PMOL HT
D	3.54	3.56	0.453	1.306
N	3.86	3.83	0.341	1.053
S	4.38	4.35	0.501	2.402
T	4.74	4.78	0.346	1.768
G	5.06	5.03	0.443	1.874
E	5.53	5.52	0.364	1.173
A	6.97	6.95	0.244	1.457
Y	8.88	8.84	0.049	0.269
R	9.91	9.88	0.028	0.505
P	10.79	10.75	0.413	1.936

PEAK ID	R.TIME (mins)	C.TIME (mins)	HEIGHT (mV)	PMOL HT
M	11.27	11.25	0.078	0.467
V	11.54	11.49	0.183	0.902
dptu	12.48	12.43	20.056	92.538
F	13.89	13.85	0.128	0.568
I	14.18	14.16	0.053	0.228
L	14.60	14.55	0.283	1.029

Supplementary Note 5: N-terminal sequencing report Tse5-CT

SequencePro™

QUIMICA DE PROTEINAS (CIB-CSIC)
Ramiro de Maeztu, 9
28040 Madrid

PNA-SQP004-F02

SAMPLE INFORMATION

Sample Name:	David Albesa_Cterminal	Std Amount:	8.000 pmols
ID Code:		Sample Amount:	0.000 pmols
		Detector Scale:	0.005 AUFS
Comments:	IIPLV		

SEQUENCER INFORMATION

Name:	PROCISE	Model Number:	494
Method:	Pulsed liquid PVDF mod	Cartridge:	D
Operator:	Javier Varela		

QUIMICA DE PROTEINAS (CIB-CSIC)
Ramiro de Maeztu, 9
28040 Madrid

Cycle 1:Blank 1

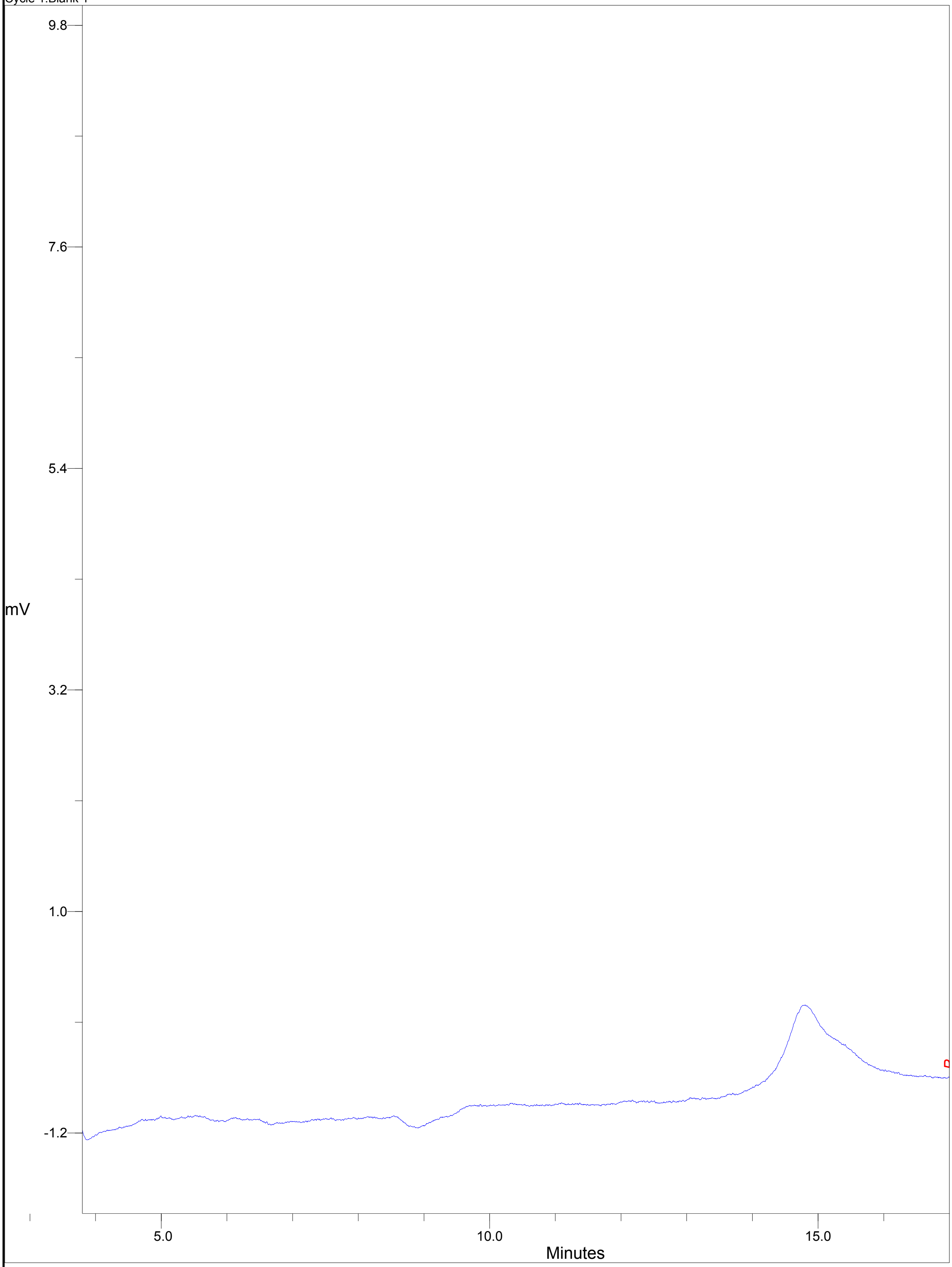

Cycle 2:

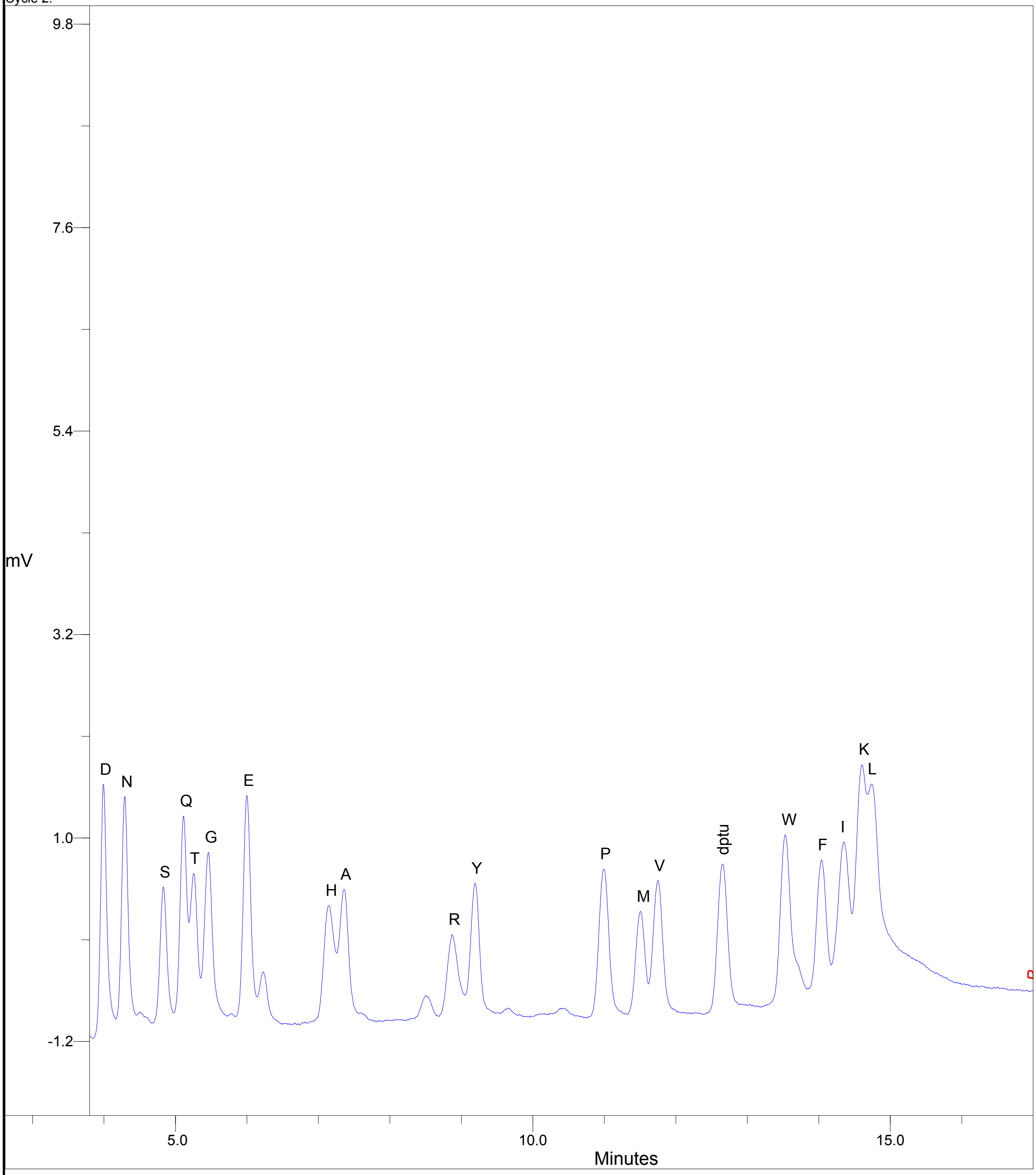

PEAK ID	R.TIME (mins)	C.TIME (mins)	HEIGHT (mV)	PMOL HT
D	3.99	3.99	2.741	8.000
N	4.29	4.29	2.591	8.000
S	4.83	4.83	1.582	8.000
Q	5.11	5.11	2.333	8.000
T	5.25	5.25	1.704	8.000
G	5.46	5.46	1.922	8.000
E	6.00	6.00	2.505	8.000
H	7.15	7.15	1.232	8.000
A	7.36	7.36	1.374	8.000
R	8.87	8.87	0.908	8.000

PEAK ID	R.TIME (mins)	C.TIME (mins)	HEIGHT (mV)	PMOL HT
Y	9.19	9.19	1.453	8.000
P	10.99	10.99	1.605	8.000
M	11.50	11.50	1.129	8.000
V	11.75	11.75	1.453	8.000
dptu	12.66	12.66	1.575	8.000
W	13.53	13.53	1.814	8.000
F	14.04	14.04	1.469	8.000
I	14.35	14.35	1.615	8.000
K	14.61	14.61	2.415	8.000
L	14.74	14.74	2.186	8.000

Cycle 3:

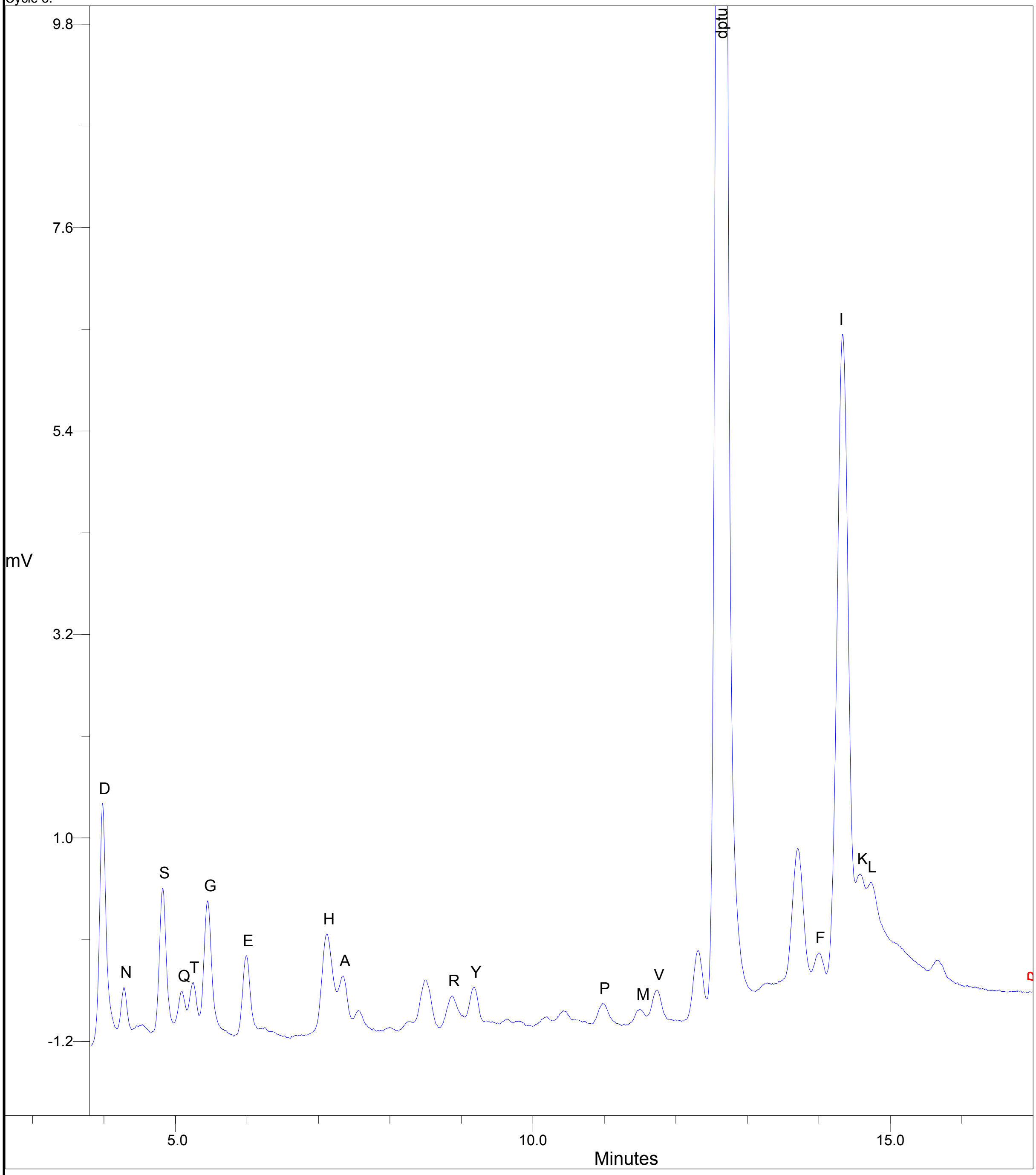

PEAK ID	R.TIME (mins)	C.TIME (mins)	HEIGHT (mV)	PMOL HT
D	3.98	3.99	2.616	7.636
N	4.28	4.29	0.612	1.888
S	4.82	4.83	1.657	8.382
Q	5.08	5.11	0.529	1.815
T	5.25	5.25	0.613	2.878
G	5.45	5.46	1.486	6.186
E	5.99	6.00	0.858	2.740
H	7.12	7.15	1.089	7.067
A	7.35	7.36	0.618	3.600
R	8.87	8.87	0.329	2.903

PEAK ID	R.TIME (mins)	C.TIME (mins)	HEIGHT (mV)	PMOL HT
Y	9.18	9.19	0.399	2.196
P	10.98	10.99	0.241	1.203
M	11.50	11.50	0.157	1.115
V	11.74	11.75	0.348	1.915
dptu	12.64	12.66	27.562	139.970
F	14.00	14.04	0.580	3.157
I	14.33	14.35	7.242	35.866
K	14.59	14.61	0.921	3.051
L	14.73	14.74	1.192	4.361

Cycle 4:

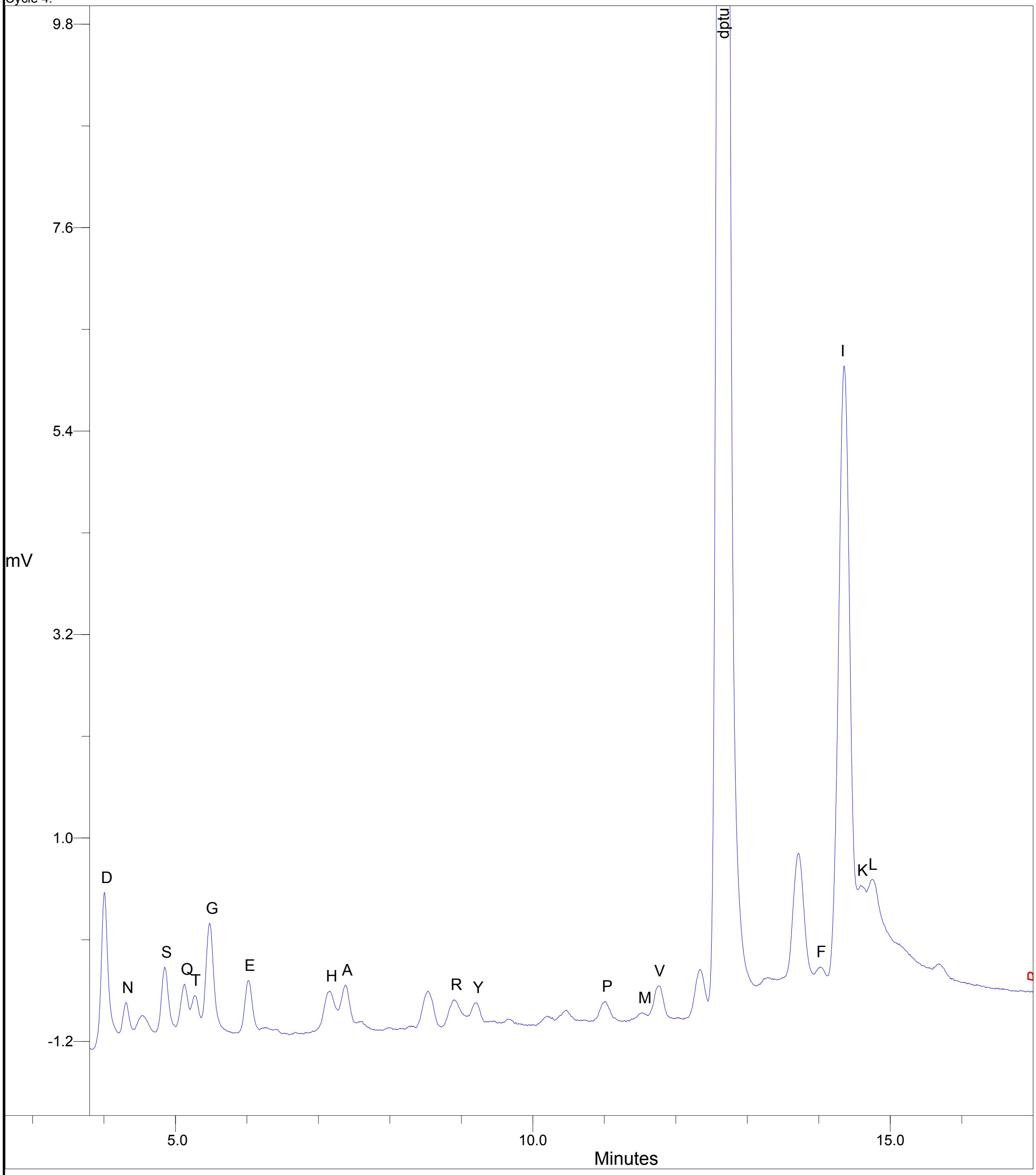

PEAK ID	R.TIME (mins)	C.TIME (mins)	HEIGHT (mV)	PMOL HT
D	4.01	3.99	1.688	4.928
N	4.30	4.29	0.480	1.481
S	4.85	4.83	0.824	4.170
Q	5.13	5.11	0.624	2.141
T	5.27	5.25	0.492	2.309
G	5.47	5.46	1.261	5.249
E	6.02	6.00	0.606	1.934
H	7.15	7.15	0.449	2.914
A	7.38	7.36	0.506	2.948
R	8.90	8.87	0.281	2.477

PEAK ID	R.TIME (mins)	C.TIME (mins)	HEIGHT (mV)	PMOL HT
Y	9.21	9.19	0.221	1.218
P	11.02	10.99	0.216	1.078
M	11.53	11.50	0.090	0.638
V	11.75	11.75	0.373	2.052
dptu	12.66	12.66	35.895	182.288
F	14.02	14.04	0.383	2.085
I	14.35	14.35	6.850	33.921
K	14.59	14.61	0.664	2.201
L	14.75	14.74	1.165	4.263

Cycle 5:

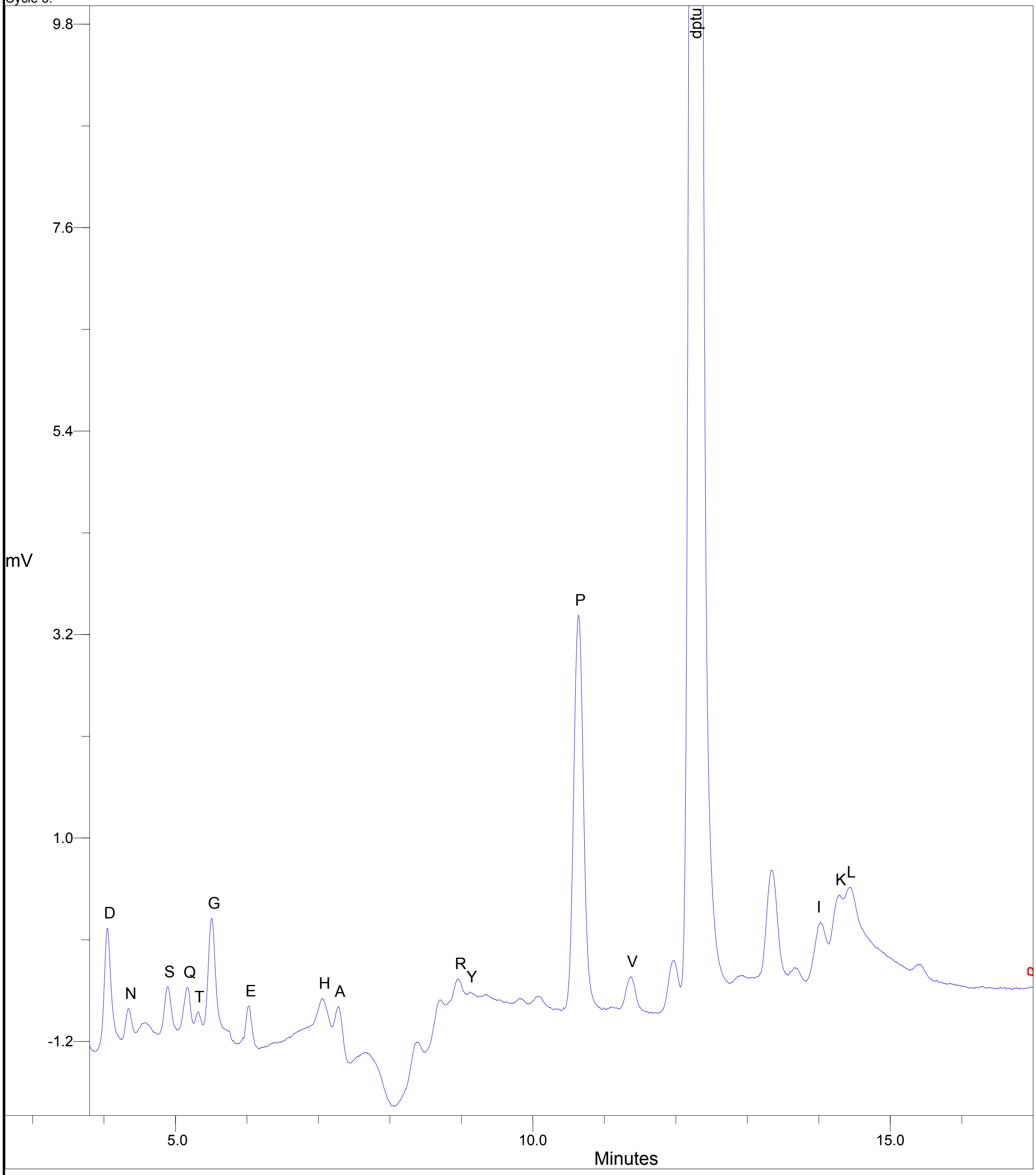

PEAK ID	R.TIME (mins)	C.TIME (mins)	HEIGHT (mV)	PMOL HT
D	4.05	3.99	1.361	3.973
N	4.35	4.29	0.532	1.643
S	4.89	4.83	0.846	4.279
Q	5.16	5.11	0.876	3.006
T	5.32	5.25	0.635	2.979
G	5.50	5.46	1.675	6.974
E	6.03	6.00	0.799	2.553
H	7.05	7.15	1.024	6.650
A	7.28	7.36	0.969	5.642
R	8.96	8.87	0.537	4.727

PEAK ID	R.TIME (mins)	C.TIME (mins)	HEIGHT (mV)	PMOL HT
Y	9.12	9.19	0.304	1.671
P	10.64	10.99	4.281	21.339
V	11.37	11.75	0.386	2.125
dptu	12.28	12.66	38.326	194.635
I	14.02	14.35	0.784	3.882
K	14.28	14.61	1.055	3.494
L	14.44	14.74	1.125	4.117

Cycle 6:

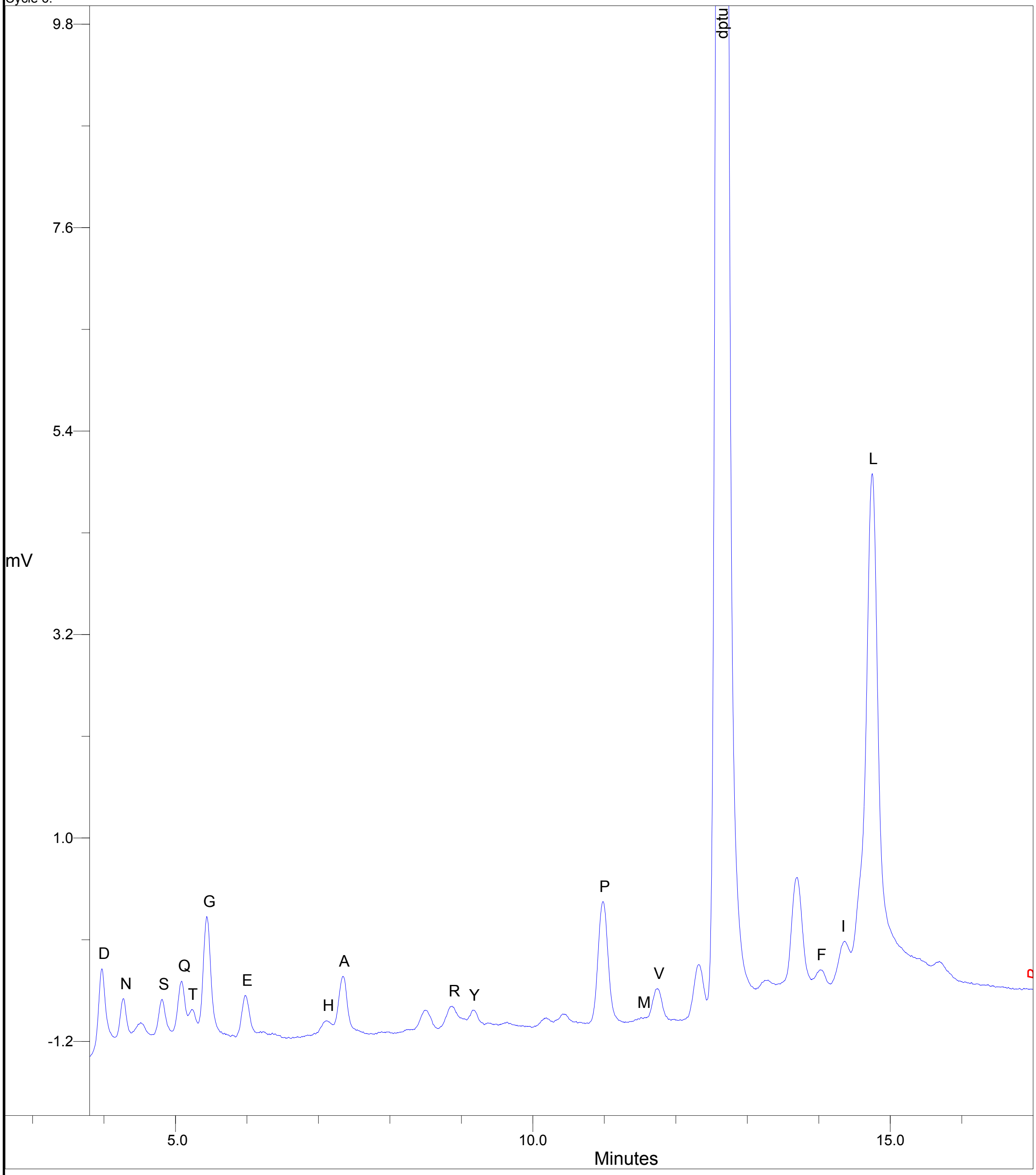

PEAK ID	R.TIME (mins)	C.TIME (mins)	HEIGHT (mV)	PMOL HT
D	3.97	3.99	0.930	2.714
N	4.28	4.29	0.569	1.757
S	4.81	4.83	0.491	2.485
Q	5.09	5.11	0.654	2.243
T	5.22	5.25	0.330	1.550
G	5.44	5.46	1.309	5.449
E	5.98	6.00	0.429	1.370
H	7.11	7.15	0.175	1.133
A	7.34	7.36	0.646	3.760
R	8.87	8.87	0.261	2.299

PEAK ID	R.TIME (mins)	C.TIME (mins)	HEIGHT (mV)	PMOL HT
Y	9.16	9.19	0.205	1.126
P	10.98	10.99	1.316	6.560
M	11.51	11.50	0.050	0.355
V	11.74	11.75	0.354	1.950
dptu	12.65	12.66	34.146	173.410
F	14.02	14.04	0.349	1.898
I	14.36	14.35	0.622	3.079
L	14.75	14.74	5.640	20.635

Cycle 7:

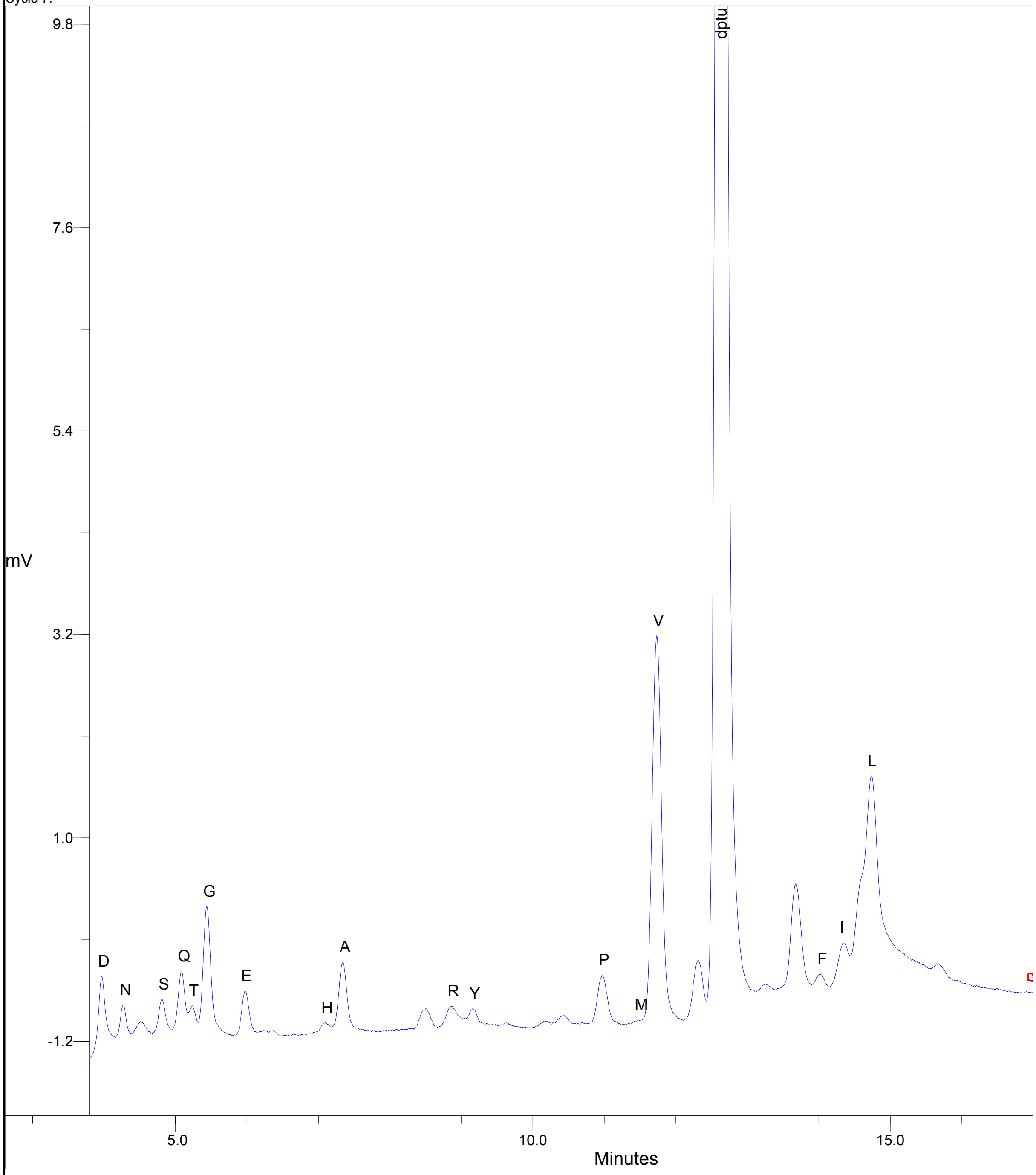

PEAK ID	R.TIME (mins)	C.TIME (mins)	HEIGHT (mV)	PMOL HT
D	3.97	3.99	0.831	2.427
N	4.27	4.29	0.435	1.342
S	4.81	4.83	0.378	1.910
Q	5.08	5.11	0.686	2.352
T	5.24	5.25	0.313	1.471
G	5.44	5.46	1.392	5.797
E	5.97	6.00	0.485	1.549
H	7.09	7.15	0.116	0.756
A	7.35	7.36	0.754	4.390
R	8.86	8.87	0.231	2.032

PEAK ID	R.TIME (mins)	C.TIME (mins)	HEIGHT (mV)	PMOL HT
Y	9.16	9.19	0.191	1.050
P	10.97	10.99	0.540	2.690
M	11.48	11.50	0.045	0.318
V	11.73	11.75	4.183	23.036
dptu	12.63	12.66	33.180	168.502
F	14.03	14.04	0.161	0.878
I	14.34	14.35	0.436	2.158
L	14.74	14.74	2.157	7.892

Supplementary references

1. van Kempen M, Kim SS, Tumescheit C, Mirdita M, Lee J, Gilchrist CLM, Söding J, Steinegger M: **Fast and accurate protein structure search with Foldseek**. *Nat Biotechnol* 2023, doi:10.1038/s41587-023-01773-0.
2. Schlegel S, Löfblom J, Lee C, Hjelm A, Klepsch M, Strous M, Drew D, Slotboom DJ, de Gier JW: **Optimizing membrane protein overexpression in the Escherichia coli strain Lemo21(DE3)**. *J Mol Biol* 2012, **423**:648–659.
3. Hood RD, Singh P, Hsu F, Güvener T, Carl MA, Trinidad RRS, Silverman JM, Ohlson BB, Hicks KG, Plemel RL, et al.: **A Type VI Secretion System of Pseudomonas aeruginosa Targets a Toxin to Bacteria**. *Cell Host Microbe* 2010, **7**:25–37.
4. Whitney JC, Beck CM, Goo YA, Russell AB, Harding BN, De Leon JA, Cunningham DA, Tran BQ, Low DA, Goodlett DR, et al.: **Genetically distinct pathways guide effector export through the type VI secretion system**. *Mol Microbiol* 2014, **92**:529–542.
5. Badaczewska-Dawid AE, Nithin C, Wroblewski K, Kurcinski M, Kmiecik S: **MAPIYA contact map server for identification and visualization of molecular interactions in proteins and biological complexes**. *Nucleic Acids Res* 2022, **50**:W474–W482.
6. Afonine P V., Klaholz BP, Moriarty NW, Poon BK, Sobolev O V., Terwilliger TC, Adams PD, Urzhumtsev A: **New tools for the analysis and validation of cryo-EM maps and atomic models**. *Acta Crystallogr D Struct Biol* 2018, **74**:814–840.
7. Letunic I, Bork P: **Interactive Tree Of Life (iTOL) v5: an online tool for phylogenetic tree display and annotation**. *Nucleic Acids Res* 2021, **49**:W293–W296.
