# Supplementary material for: Structural and functional insights into the delivery of a bacterial Rhs pore-forming toxin to the membrane
Source: Nat Commun. 2023 Nov 28;14:7808. doi: 10.1038/s41467-023-43585-5 (PMC10684867; doi:10.1038/s41467-023-43585-5)
Supplement: Supplementary file 3 — Description of Additional Supplementary Files [file 41467_2023_43585_MOESM3_ESM.pdf]

Description of Additional Supplementary Files:

Supplementary Data 1: Tse5 homologues identified by Foldseek.

Supplementary Data 2: Tse5 sequence derived for structural and biophysical studies.

Supplementary Data 3: List of essential materials employed in this study.

Supplementary Movie 1: Movie showing the MD trajectory of the Tse5-membrane simulation. Visualisation of the 1μs Molecular Dynamics simulations conducted using GROMACS 2020.4. The molecular system was assembled with CHARMM-GUI in order to generate an atomic interfacial membrane binding model. CHARMM-GUI applied the PPM Server to position Tse5 in the membrane. The movie shows Tse5 binds to the surface of the membrane, showing major conformational changes in Predicted Helical Region 1 and Helical Region 2.
