# Supplementary material for: Structural and functional insights into the delivery of a bacterial Rhs pore-forming toxin to the membrane
Source: Nat Commun. 2023 Nov 28;14:7808. doi: 10.1038/s41467-023-43585-5 (PMC10684867; doi:10.1038/s41467-023-43585-5)
Supplement: Supplementary file 5 — Dataset 2 [file 41467_2023_43585_MOESM5_ESM.zip › Supplementary Data 2.docx]

# **Tse5 sequence derived for structural and biophysical studies.**

MMGSSHHHHHHHHHSSGENLYFQGGSMSGLPVSHVGEKVSGGVISTGSPTVHVGSSAVGLADRVSACVPLVGKPVNPMLGSKLLPEEVDFALAAPDTFTFARGYLSSNPRIGRLGRGWWLPGESMHLELSEDACVLVDAQGRRIGFPALAPGAQHYSGSEELWLRRGGSSGGEAQAWRGRWAAVPAELQTQEGSVLVLSGHSYLHFQRCPDGIWRLQASFGRAGYRTEFRWSGRGLLTGVRDSAGRSYALVYQQACEPSEGDDGLRLFGVILASHDGPPPDYIDPQSPGLDWLVRYQFSDSGDLIAVRDRLGQVVRVFAWREHMLVAHGEPGGLEVRYEWDVHAPHGRVVKQIEAGGLTRTFRYLRDATEVSDSLGRVERYEFAGEGGQRRWTALVRADGSRSEFDYDLFGRLVAMRDPLGRETRRRRDGQGRMLEEESPGKARYRKRVDEETGLLVELEDAMQRRWTFERDERGNATTVRGPAGSTRYAYEDPRLPDRPTRIVDPRGGERRLEWNRFGLLAALTDCSGQVWRYDYDNEGRLVASSDPLGQLTRRRYDPLGQLIGLELADGSALSYEYDALGRQTRIADAEGHATLFSWGHGDLLARVSDAGGGELSYLHDEAGRLVALTNENGVQAQFRYDLLDRLVEETGFDGRRQRYRYNAADELIAREDADGRETTYAYDRDGRLASIRVPATEHAPALVERYRWLADGRLASAGGADCEVRYTYDEVGNLRLESQVHADGWVYSVEHSHDALGVRQTSRYGDAPPVAWLTYGPGHLHGALVGAVELAFERDALHREVRRDARRDGQDDALFTQERQHAPLGRLQRSRLRLAGGFDWQRGYRYDGLGQLVGIDDNQYPSVRYEYDLGGRLLASRRAGAAASTYRYDAAGNRLEGVGEHAREDARQAFAENELYRSGFSRSETRASQAGEGPARWAGNRVERIAGNRYRFDALGNLVERIGADGERLRLAYDGAQRLVHLTRDYADGTRLEARYRYDALSRRIAKVVLRDGVEQQVRFGWDGDRQCAEAFARELRTTVHEPGGFVPLLRLEQACEPDPPELLQLRQAFAAEGQPLPAQCVPALGEARIAFFHTDHLGTPLQLSDERGQLRWQGVPDDWRAVAPERQPGAQPIRFQGQYHDEESGLYYNRYRYYLPEAGRYASQDPLGLGGGPNPYAYALNAPTLAYDPTGLIIPLVVIGAFAARAAIGAALGAGIELGMQTGKQVLGQMKDNWDSDRDLTDIKWKCIDINWKHVGASAAIGTVAPGMLSTGKTVVQSAKAIRTLSGQAANTANRAAKLAARKAAHADTIKKAVATQAAWQTGKQIVKCPLKDEEEECPPQ*

His-tag

Tse5-NT

Tse5-CT

NT-plug, CT-plug
