# Supplementary material for: Structural and functional insights into the delivery of a bacterial Rhs pore-forming toxin to the membrane
Source: Nat Commun. 2023 Nov 28;14:7808. doi: 10.1038/s41467-023-43585-5 (PMC10684867; doi:10.1038/s41467-023-43585-5)
Supplement: Supplementary file 8 — Reporting Summary [file 41467_2023_43585_MOESM8_ESM.pdf]

nature portfolio

Corresponding author(s): Iban Ubarretxena-Belandia and David Albesa-Jové

Last updated by author(s): Nov 2, 2023

Reporting Summary

Nature Portfolio wishes to improve the reproducibility of the work that we publish. This form provides structure for consistency and transparency in reporting. For further information on Nature Portfolio policies, see our Editorial Policies and the Editorial Policy Checklist.

Statistics

For all statistical analyses, confirm that the following items are present in the figure legend, table legend, main text, or Methods section.

n/a	Confirmed	
☐	☒	The exact sample size (*n*) for each experimental group/condition, given as a discrete number and unit of measurement
☐	☒	A statement on whether measurements were taken from distinct samples or whether the same sample was measured repeatedly
☐	☒	The statistical test(s) used AND whether they are one- or two-sided<br>*Only common tests should be described solely by name; describe more complex techniques in the Methods section.*
☒	☐	A description of all covariates tested
☒	☐	A description of any assumptions or corrections, such as tests of normality and adjustment for multiple comparisons
☐	☒	A full description of the statistical parameters including central tendency (e.g. means) or other basic estimates (e.g. regression coefficient) AND variation (e.g. standard deviation) or associated estimates of uncertainty (e.g. confidence intervals)
☐	☒	For null hypothesis testing, the test statistic (e.g. *F*, *t*, *r*) with confidence intervals, effect sizes, degrees of freedom and *P* value noted<br>*Give P values as exact values whenever suitable.*
☐	☒	For Bayesian analysis, information on the choice of priors and Markov chain Monte Carlo settings
☒	☐	For hierarchical and complex designs, identification of the appropriate level for tests and full reporting of outcomes
☒	☐	Estimates of effect sizes (e.g. Cohen's *d*, Pearson's *r*), indicating how they were calculated

Our web collection on statistics for biologists contains articles on many of the points above.

Software and code

Policy information about availability of computer code

Data collection	CryoEm data collection was performed in a in a 300 kV ThermoFisher Titan Krios transmission electron microscope with a Falcon 4 direct electron detector at the UK's National Electron Bio-imaging Centre. Synchrotron SAXS data were collected on an EigerX 4M (Dectris) pixel detector at Diamond Light Source B21 beamline (UK). Electrophysiology currents were measured with an Axopatch 200B amplifier (Molecular Devices, Sunnyvale, CA) in the voltage-clamp mode. Langmuir–Blodgett balance data were collected using a DeltaPi-4 Kibron tensiometer (Helsinki, Finland). QCM-D experiments were recorded with a Q-SENSE E4 system (Q-Sense, Sweden).
Data analysis	CryoEM data processing, analysis, model building and refinement were performed with CryoSPARC v.4, CPPEM v 1.6.0 package, Coot 0.9.8.91 and Phenix 1.20.1-4487 package. Validation was performed with MolProbity and the PDB validation server. SAXS data were processed using the program packages ScÅtterIV and PRIMUS 3.1. Electrophysiology traces were analysed using pClamp 10 software (Molecular Devices, Sunnyvale, CA). Monolayer experiments and QCM-D experiments were analysed using GraphPad Prism v.9.5

For manuscripts utilizing custom algorithms or software that are central to the research but not yet described in published literature, software must be made available to editors and reviewers. We strongly encourage code deposition in a community repository (e.g. GitHub). See the Nature Portfolio guidelines for submitting code & software for further information.

Data

Policy information about availability of data

All manuscripts must include a data availability statement. This statement should provide the following information, where applicable:

- Accession codes, unique identifiers, or web links for publicly available datasets
- A description of any restrictions on data availability
- For clinical datasets or third party data, please ensure that the statement adheres to our policy

The authors declare that source data supporting the findings of this study are available within the paper and its supplementary information files. Atomic coordinates of the Tse5 structure have been deposited in the Protein Data Bank (accession code id: 8CP6). The cryo-EM map is available from the Electron Microscopy Data Bank (accession code id: EMD-16778 [https://www.ebi.ac.uk/emdb/EMD-16778]). Supplementary Data 1-4 are available as supplementary files and contain a list of Tse5 homologues identified by Foldseek, the Tse5 protein sequence derived for structural and biophysical studies, a list of essential materials employed, and data plotted in Figures 3-4, respectively. Supplementary Movie 1 shows the 1s MD trajectory of the Tse5-membrane simulation. Supplementary Notes 1-5 are available in the Supplementary Information file and contain the bioinformatic analysis of Tse5 homologues (Note 1), LC-ESI-MS report (Note 2), and the N-terminal sequencing reports for Tse5 (Notes 3-5). Uncropped and unedited gel images are included in Supplementary Fig. 11.

Research involving human participants, their data, or biological material

Policy information about studies with human participants or human data. See also policy information about sex, gender (identity/presentation), and sexual orientation and race, ethnicity and racism.

Reporting on sex and gender	n/a
Reporting on race, ethnicity, or other socially relevant groupings	n/a
Population characteristics	n/a
Recruitment	n/a
Ethics oversight	n/a

Note that full information on the approval of the study protocol must also be provided in the manuscript.

Field-specific reporting

Please select the one below that is the best fit for your research. If you are not sure, read the appropriate sections before making your selection.

[x] Life sciences   [ ] Behavioural & social sciences   [ ] Ecological, evolutionary & environmental sciences

For a reference copy of the document with all sections, see nature.com/documents/nr-reporting-summary-flat.pdf

Life sciences study design

All studies must disclose on these points even when the disclosure is negative.

Sample size	No sample size calculation was performed. Sample sizes were chosen depending on the technical requirements of each technique employed. Thus, for Cryo-EM studies, 10,244 movies were recorded, which resulted in 323,963 protein particles being used for 3D reconstruction. For size exclusion chromatography-Small-angle X-ray scattering (SEC-SAXS), proteins were injected into the Shodex KW-403 column at 2.5 mg/ml to obtain sufficient signal. Langmuir–Blodgett balance experiments were performed at 0.4 µM of sample to obtain sufficient signal. Electrophysiology experiments were performed adding 168 nM toxin to ensure that it reaches the lipid bilayer and forms pores. For bacterial competition assays, a sample size of 3 was chosen due to significant and reproducible differences between groups. Identical sample sizes have been used for similar experiments published previously.
Data exclusions	No data were excluded from the analysis
Replication	All the experiments were replicated at least 3 times to ensure the reproducibility. Findings could be realiably reproduced. Bacterial competition assays were repeated in three independent experiments, with three independent cultures in each experiment.
Randomization	Randomization is not relevant to our study because it is not a clinical trial or a research with different groups/participants.
Blinding	Blinding is not relevant to our study because it is not a clinical trial or a research with different groups/participants.

Reporting for specific materials, systems and methods

We require information from authors about some types of materials, experimental systems and methods used in many studies. Here, indicate whether each material, system or method listed is relevant to your study. If you are not sure if a list item applies to your research, read the appropriate section before selecting a response.

Materials & experimental systems

n/a	Involved in the study
☒	☐ Antibodies
☒	☐ Eukaryotic cell lines
☒	☐ Palaeontology and archaeology
☒	☐ Animals and other organisms
☒	☐ Clinical data
☒	☐ Dual use research of concern
☒	☐ Plants

Methods

n/a	Involved in the study
☒	☐ ChIP-seq
☒	☐ Flow cytometry
☒	☐ MRI-based neuroimaging
